# Understanding Polymer Electrodeposition and Conducting Polymer Modified Electrodes Using Electrochemistry, Spectroscopy, and Scanning Probe Microscopy

**DOI:** 10.1021/acs.jchemed.3c00656

**Published:** 2023-09-13

**Authors:** Jessica M. Bone, Judith L. Jenkins

**Affiliations:** Department of Chemistry, Eastern Kentucky University, Richmond, Kentucky 40475, United States

**Keywords:** Upper-Division Undergraduate, Graduate Education, Analytical Chemistry, Polymer Chemistry, Interdisciplinary/Multidisciplinary, Hands-On Learning, Decision Making, Electrochemistry, Materials Science, Polymerization, Surface Science, UV−Vis Spectroscopy

## Abstract

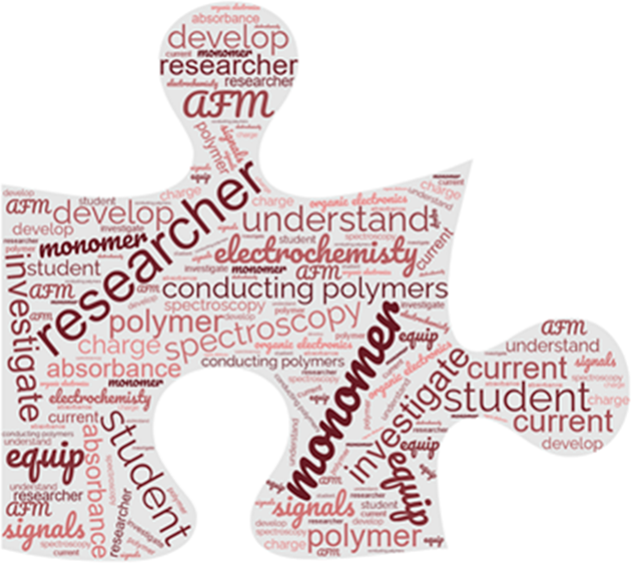

Conducting polymers are critically important materials in organic electronic platforms relevant to sustainability (organic photovoltaics and organic light-emitting diodes) and wearable electronics (organic electrochemical transistors). However, most chemistry students do not receive formal training in the fundamental properties and extensive characterization of these fascinating materials. Described here are four scaffolded learning modules adapted from the primary literature and designed to build the fundamental understanding and practical skills necessary for productive contribution to emerging research in the field of conducting polymers and conducting polymer modified electrodes (CPMEs). These activities were performed by first-year chemistry graduate students and have been used in the lab to orient and equip new student researchers with the electrochemical, spectroscopic, and spectroelectrochemical skillsets central to working in CPMEs. First year master’s students and undergraduate student researchers worked individually to complete data collection, analysis, and interpretation over three 4 h periods with additional time for sample preparation and imaging. Alternatively, one or more of these modules can be adapted and performed by pairs or groups of three over two 4 h lab periods as part of an undergraduate course such as instrumental analysis, polymers, and macromolecules, or as a capstone experience; instructions for these and other modifications are as described herein. If lab equipment and/or available time are limiting factors, sufficient sample data are provided for use as dry laboratories. Through completion of these modules, student researchers learn how to build chemically rational explanations for the electrochemical and spectroscopic signals, to collectively examine data from multiple complementary characterization techniques, and to extract enabling structure–property relationships, all while coming to see themselves as researchers and members of a worldwide scientific community.

Enabled by the discovery and development of conducting polymers (CPs),^1^ electrodes modified with CPs have been incorporated into sensors, actuators, lighting, energy conversion platforms, and other organic electronics.^2−4^ The widespread utility of conductive polymer modified electrodes (CPMEs) arises from the versatile optical and electrical properties of easily processed CPs. Well-studied CPs include polyacetylene, polyaniline (PANI), poly(3,4-ethylenedioxythiophene) (PEDOT), polyparaphenylenevinylene (PPV), polypyrrole, polythiophenes, and derivatives of these, where the chemistry of π-conjugated monomeric units can be used to tune electrical and optical properties of a CPME for a given application.^2^ See for example a recent review by Ibanez et al.^3^ Poly(alkylthiophenes) (PATs) are a particularly interesting family of CPs, where alkyl substituents on a thiophene serve to increase the solubility of the resulting polymers. PATs are typically synthesized via Grignard metathesis to yield high molecular weight polymers with limited solubilities in organic solvents.^5^ A well-studied PAT, poly(3-hexylthiophene), is a promising material in photovoltaic applications when blended with fullerene derivatives like [6,6] phenyl-C_61_-butyric acid methyl ester (PCBM), spincast onto a transparent conducting substrate, and thermally annealed to yield highly ordered, closely packed P3HT:PCBM thin films with desirable optoelectronic properties.^6^ Spectroscopic and spectroelectrochemical characterization techniques are necessary to understand the relevant PAT structure–property relationships. CPs also show promise as organic electrochemical transistors (OECTs) with applications of artificial synapses^7^ and other wearable electronics.^8^ A recent report describes use of PAT side chains to tune conductivity in polythiophene-based OECTs.^9^ Applications in wearable electronics and biosensing require understanding the properties of porous CPMEs in contact with electrolytic solutions via electrochemical characterization techniques relevant to OECT applications.^10^

CPMEs represent rich teaching and learning opportunities, where fundamental chemistry of CPs and materials characterization can be learned in societally relevant context.^4^ Reports in this *Journal* allow students to synthesize and/or characterize CPs.^11−15^ Laboratory experiences to fabricate, test, and optimize working devices like sensors and actuators,^16,17^ batteries,^18,19^ photovoltaics,^20^ conductive textiles,^21^ and electrochromics^22^ are widely available. Together, these experiments represent robust introductory experiences for students, exposing them to CPs and providing real-world examples of their functionalities in CPMEs.

There are fewer examples of laboratory experiences providing multifaceted exposure to CPs, their uses in CPMEs, and complementary techniques for characterization of these materials and interfaces. Cortes et al.^17^ combine electrodeposition of polypyrrole with demonstrations of the polymer film’s performance as an actuator or sensor—relating molecular-level changes to observed function. Sherman et al.^13^ employ spectroelectrochemistry to illustrate electrochromic properties of electrodeposited polyaniline films. Seng et al.^15^ demonstrate varied conductivities of electrodeposited polyaniline as a function of deposition solution compositions. Electropolymerization and polymer electrodeposition are used to fabricate CPMEs,^11,13,15−18^ but experiments describing electropolymerization mechanistically have not yet been reported. Similarly, several reports in this *Journal* facilitate meaningful mechanistic understanding of metal electrodeposition,^23−25^ but to our knowledge, there are no analogous experiences addressing the nucleation and growth processes occurring during polymer electrodeposition, motivating this work.

Described here are four learning modules that build fundamental understanding and practical skills necessary for productive contribution to emerging research in the field of CPs and CPMEs, adapted from primary literature.^26−28^ Student researchers electropolymerize and electrodeposit model poly(alkylthiophene) thin films from solutions of the corresponding monomer, indirectly observing polymerization through the evolution of electrochemical and spectroscopic signals. Atomic force microscopy, used to collect topographical images of the CPMEs, allows student researchers to visualize the electrode surface as polymer nucleation and growth proceed. Spectroelectrochemical characterization yields a deeper understanding of CP optical and electrical properties, while use of solution redox probes reveals functional impacts of CP subpopulations, together illustrating structure–property relationships. Importantly, these modules present a realistic example of reliance on multiple complementary characterization techniques when understanding complex systems. These modules build a molecular-level understanding of chemistry enabling functionality of CPs and their uses in CPMEs while also developing the next generations of researchers and innovators. A collection of detailed procedures, structured documentation for each module, in- and postmodule questions, and rubrics are provided in Supporting Information (SI), while institutional context, pedagogy, assessment, and important background information are summarized here alongside representative data.

## Activity Design and Goals

### Institutional Context

Modules were designed for first-year chemistry graduate students and advanced undergraduate independent researchers, assuming the completion of undergraduate instrumental analysis. These modules were used to orient new MS students to the group’s research. Modules can be completed in sequence or as stand-alone activities. Modules may be useful for undergraduate teaching laboratories in instrumental analysis, polymers and macromolecules, or materials characterization. Regardless of curricular preparation, familiarity with bench chemistry (using appropriate glassware, making solutions) and general laboratory safety (personal protective equipment, responses to spills and other accidents, appropriate disposal of waste) is necessary. Specific hazard considerations are provided in SI.

### Implementation Description

MS students completed modules independently in three 4 h lab periods with additional time for preparation, imaging, and analysis. Module 1 required an hour of preparation prior to data collection and 4 h for experiments. For Module 2, each sample setup took 15–20 min, and image collection required ∼12 h. Modules 3 and 4 required 4 h each for data collection and 1–2 h for analysis and interpretation.

When adapting for undergraduates, it is recommended that students complete modules in pairs over two 4 h lab periods. Rather than all groups completing all modules, it is recommended that all groups complete Module 1 in the first lab period. The remaining modules may be assigned to specific groups and completed simultaneously during the second lab period. Scanning probe microscopy can be expensive if it is not already available. Given this and the time required for imaging, use of images in figures when adapting Module 2 for undergraduates is recommended. Module 3 can be shared, with one group completing part A and one group completing part B. If time and/or cost are limiting, use of representative data provided here in dry laboratories or other critical thinking exercises is encouraged.

### Student Prior Knowledge

The graduate (MS) students completing the modules had obtained undergraduate chemistry degrees with coursework 2 semesters of general chemistry with laboratories, 2 semesters of organic chemistry with laboratories, quantitative analysis (1 semester) and instrumental analysis (1 semester) both with laboratories, and at least one semester of biochemistry, inorganic, and physical chemistry. When adapting these modules for undergraduates, ensure students’ prior knowledge includes familiarity with analytical techniques (electrochemistry, spectroscopy) and molecular properties and phenomena (electron transfer reactions, electronic transitions, and molecular orbitals). No prior exposure to atomic force microscopy, conducting polymers, or organic electronics are needed.

#### Electrochemistry

Students should understand electron transfer reactions (oxidation, reduction, half reactions) at the level commonly encountered in general chemistry. Students should have at least one experience using a 3-electrode electrochemical cell to perform cyclic voltammetry and identifying the Faradaic and non-Faradaic current on the resulting voltammogram. If students have not encountered electrochemical techniques, reading the guide to cyclic voltammetry described by Dempsey^29^ and completion of the modules within are suggested prerequisite experiences.

#### UV–Vis Absorbance Spectroscopy

Students should understand fundamentals of UV–vis absorbance spectroscopy including the basic components of a spectrophotometer, use of the Beer–Lambert law, and be aware of vibrational and electronic transitions in molecules monitored with absorbance spectroscopy. Students should have at least one experience using a UV–vis spectrophotometer and interpreting an absorbance spectrum.

#### Polymer Chemistry

Students should be able to define monomer and polymer and recognize polymerization conceptually (not necessarily mechanistically). Students should be able to compare molecular orbitals (aliphatic molecules, for instance) and conjugated p-electron systems (aromatic molecules and electronic polymers). Students should be familiar with band theory as it relates to solids and should be familiar with using band theory to depict the properties of insulators, semiconductors, and conductors.

#### Required Prelaboratory Readings

Works by Heinze^30,31^ were used to introduce students to thiophene electropolymerization and electrodeposition. A theoretical description of semiconducting polymers in organic electronics by Luscombe^4^ introduced students to structure–property relationship observed in polymer systems that afford their functionality in electronic devices. Interpretation of poly(alkylthiophene) optical and electrical properties measured in the modules was supported using previously published work.^28^ In this setting, students read and informally discussed this literature. When adapting for undergraduates, use of reading guides and more structured documentation, all matched to students’ experiences interpreting literature and the course goals, are recommended.

### Learning Outcomes

Through these modules, students build practical skills, develop rich conceptual understanding, and grow into contributing researchers. Activities are designed around three key goals. First, students will learn and understand laboratory and characterization techniques. Specifically, they will be able to

- Electrochemically fabricate conducting polymer modified electrodes (CPMEs) (Goal Component 1.1).
- Characterize CPMEs using cyclic voltammetry, AFM (topography), absorbance spectroscopy, and redox-active probe molecules in solution; process and interpret these data (Goal Components 1.2–1.4; one component for each technique).
- Select and apply appropriate characterization techniques when analyzing CPMEs (Goal Component 1.5).

Second, students will learn how to develop chemically rational explanations for/from measured signals evidenced by their abilities to

- Explain electrochemical signals observed from molecular-level perspectives, clearly articulating processes that give rise to measured signals in the context of electropolymerization, electrodeposition, and behaviors as a function of polymer subpopulations (Goal Component 2.1).
- Relate electrochemical data (current density, charge density) to the amount of polymer electrodeposited (Goal Component 2.2).
- Connect electrochemical data and topographical images to construct molecular-level understanding of polymer nucleation and growth processes from evidence (Goal Component 2.3).

Third, students will learn how to evaluate and leverage structure–property relationships in electroactive polymers. They will:

- Use spectroscopic and spectroelectrochemical data to identify and understand neutral and charged polymer subpopulations (Goal Component 3.1).
- Use solution redox probes to examine CPME conductivity as a function of PAT subpopulation (Goal Component 3.2).
- Realize they can alter relative concentrations of neutral and charged PAT subpopulations electrochemically to meet application design criteria (Goal Component 3.3).

### Pedagogical Considerations

Module designs were informed by constructivist pedagogical considerations and using a backward design process. That is to say, the learning outcomes were identified and then activities were designed to facilitate those goals. Rather than separate procedures from data processing and analysis, structured documentation and questions for analysis and interpretation were integrated into procedures as scaffolding to facilitate learning, and knowing students complete these modules independently, with minimal input from instructors. Newer learners are known to ascribe meaning to features that may not be particularly meaningful, and structured documentation supports students learning to think chemically, making sense of large data sets by highlighting key features or properties.^32^ Structured documentation (tables for recording peak potentials, absorbance values) draws students’ attention to meaningful data points lest students get overwhelmed by the magnitude of data and/or distracted by attempts to ascribe meaning to less relevant features.^33^ Questions embedded in procedures serve as scaffolding and help students bridge gaps between their awareness of analytical techniques and more rich and nuanced understandings of how and why a given technique can be employed to provide information about a chemical system.^32,33^ The prompts to develop particle-level depictions of the chemical processes that give rise to analytical signals promote the development of mental models and representational competency.^34,35^

In parallel, efforts to engage students’ intrinsic motivations were made, knowing that participants were considering STEM careers. Drawing on evidence that understanding of societal relevance increases retention in STEM,^36,37^ chemical systems applicable to organic electronics were chosen, as organic electronic devices show promise in fields of sustainable energy conversion and storage,^6^ biomonitoring,^3^ and a range of monitoring and sensing platforms.^10^ Students experienced the application of analytical techniques to address pressing challenges of personal relevance. Procedures were written for independent student use, meaning without assistance from an instructor, to further students’ perceptions of themselves as scientists.^36,38,39^ Given these considerations, and because in this context, students were not earning letter grades for their participation in the experiences, standards-based assessment was used.^40−42^

### Assessment of Learning Outcomes and Evidence of Efficacy

Standards for proficiency were developed using the components of Goals 1–3; students were assessed for proficiency using rubrics (SI). To date, two MS students and two undergraduate students in independent research experiences have completed parts or all modules. Upon graduation, these students either are employed vocationally as chemists or are pursuing chemistry doctoral degrees. Independent characterization and deep analysis of results with respect to a specific chemical system or application are priorities. As such, proficiency in all Goal 1 and Goal 2 components was required and evaluated on a satisfactory/unsatisfactory basis. The extent of proficiency required on Goal 3 depended on a student’s future research and was similarly evaluated.

Students readily demonstrated proficiency on all Goal 1 components; this was expected, given students’ previous exposure to analytical techniques during undergraduate programs. Qualitative increases in confidence were observed. These were attributed in part to the increased practice with techniques and increased chemical understanding. These modules allowed students to build on what they had learned previously and to apply fundamental conceptual knowledge to a different chemical system. Increased confidence was also attributed to students’ pursuit of competencies and skills necessary to become independent scientists. We suggest that these modules played a part in allowing students to see themselves as scientists, able to use their skillsets and conceptual understanding to address societal challenges, as evidenced by their career trajectories.

Students struggled to immediately demonstrate proficiency on Goal 2 components and were allowed repeated attempts to demonstrate proficiency. Goal Component 2.1 was particularly challenging, as these modules were students’ first exposure to CPs and multiple polymeric subpopulations. Iterative comparison of experiments performed, data collected, and relevant literature (in particular, that of Heinze) ultimately yielded proficiency in Goal 2. Similar iterative work yielded proficiencies in Goal 3.

Correlating standards to course grades or other metrics depends on local implementation goals. For instance, in undergraduate instrumental analysis, exposure to multiple techniques and exposure to multiple chemical systems are high priorities. As such, recommended proficiencies for a passing grade and a minimum number of proficiencies for earning an A, B, C, or D were identified (SI). For example, to earn a passing grade (C or higher), a demonstration of proficiency on components 1.1, 1.2, 2.1, and 3.1 is recommended, with additional proficiencies required to earn an A or B.

## Background and Theory

### Electropolymerization and Electrodeposition

**Figure 1:**
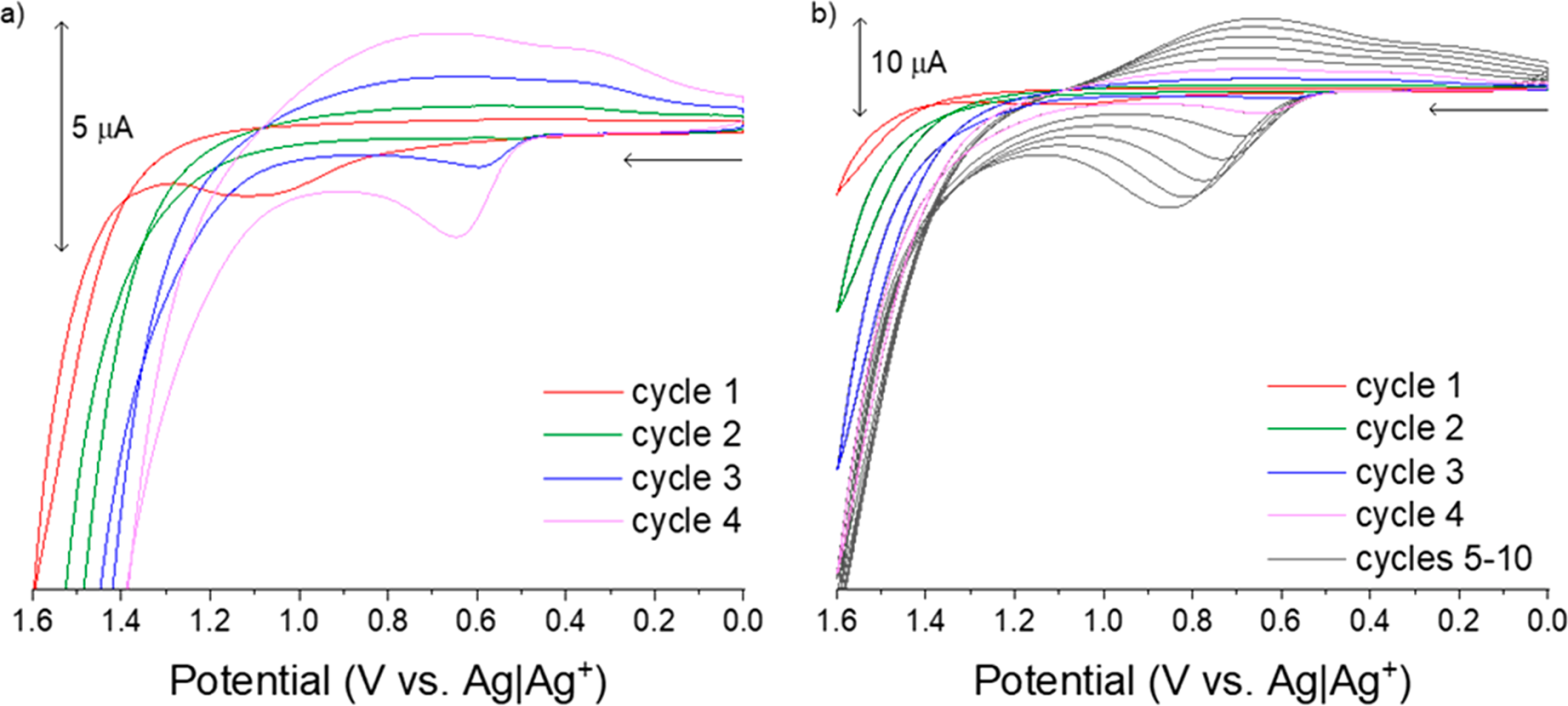
Electropolymerization and electrodeposition of poly(alkylthiophene) via cyclic voltammetry (ν = 100 mV/s, 10 cycles). The first 4 potential cycles (a) yield oxidation of monomers and oligomer formation; by cycle 3 evidence of nucleation appears. Voltammograms for all 10 cycles (b) illustrate the nucleation and growth of the polymer. The electrolyte was tetrabutylammonium hexafluorophosphate (TBAPF_6_, 0.1 M in acetonitrile) while the monomer solution was 3-dodecylthiophene (3DDT, 50 mM monomer and 0.1 M TBAPF_6_ in acetonitrile). The working electrode was a functionalized planar indium tin oxide thin film on a glass substrate; the electrode area was defined by an O-ring with an area of 0.76 cm^2^. The counter electrode was an ITO substrate oriented parallel to the working electrode, and the reference electrode was Ag|Ag^+^ (10 mM AgNO_3_, 0.1 M TBAPF_6_ in acetonitrile). See Supporting Information Module 1 for additional experimental details. If performing a dry lab, use this figure as part of Module 1.

Electrochemical polymerization offers a simple and scalable way to prepare polymeric films within a short reaction time under mild reaction conditions. This technique is limited to conductive substrates. Electropolymerization and electrodeposition of a poly(alkylthiophene) via cyclic voltammetry are shown in Figure 1.

The mechanistic steps of electropolymerization are well-described by Heinze et al.^30,31^ and summarized here for context, beginning from a monomeric solution in an electrochemical cell. Note that all potentials are with respect to a nonaqueous Ag|Ag^+^ reference electrode. As the potential is swept oxidatively to a potential at which the neutral monomers near the electrode surface are oxidized, giving rise to the Faradaic current observed (Figure 1a, red) near +1.0 V. When oxidized monomers collide with sufficient energy and orientation, they react to form dimers and other short oligomers. As the potential sweep is reversed, monomer oxidation halts, and diffusion processes replenish the relative concentration of neutral monomers near the electrode surface. Diffusion of dimers is expected to be slower than that of the monomer, so some concentration of dimers also remains near the electrode surface.

On the second potential cycle (Figure 1a, green), the so-called nucleation loop is observed evidenced by a Faradaic current near +0.8 V; it is energetically easier to oxidize dimers relative to monomers because of the increased conjugation length, yielding a Faradaic current at less positive potentials than those observed in the first cycle. Oxidized dimers collide to form tetramers and other oligomers, while additional oxidized monomers are generated when the potential is sufficiently positive. Together this yields a heterogeneous population of electroactive neutral and oxidized monomers, dimers, and longer oligomers near the electrode surface.

Polymer electrodeposition occurs when oligomers are too long to remain soluble and precipitate onto the nearest surface (Figure 1a, blue), commonly the working electrode. Poly(alkylthiophenes) are typically electrodeposited from an organic solvent to accomplish the desired solubility of monomers and insolubility of longer polymer chains; most alkylthiophenes are insoluble in water. These precipitated oligomers form nucleation sites from which longer polymer chains grow, as they are the longest oligomers (most easily oxidized, thermodynamically) and are confined to the working electrode surface as evidenced by Faradaic current at +0.65 V (the least positive oxidation current observed in the first 10 cycles). In subsequent oxidative sweeps, electropolymerization and electrodeposition occur, evidenced by increases in peak currents and the shift in polymer oxidation to less-positive potentials (Figure 1b).

**Figure 2:**
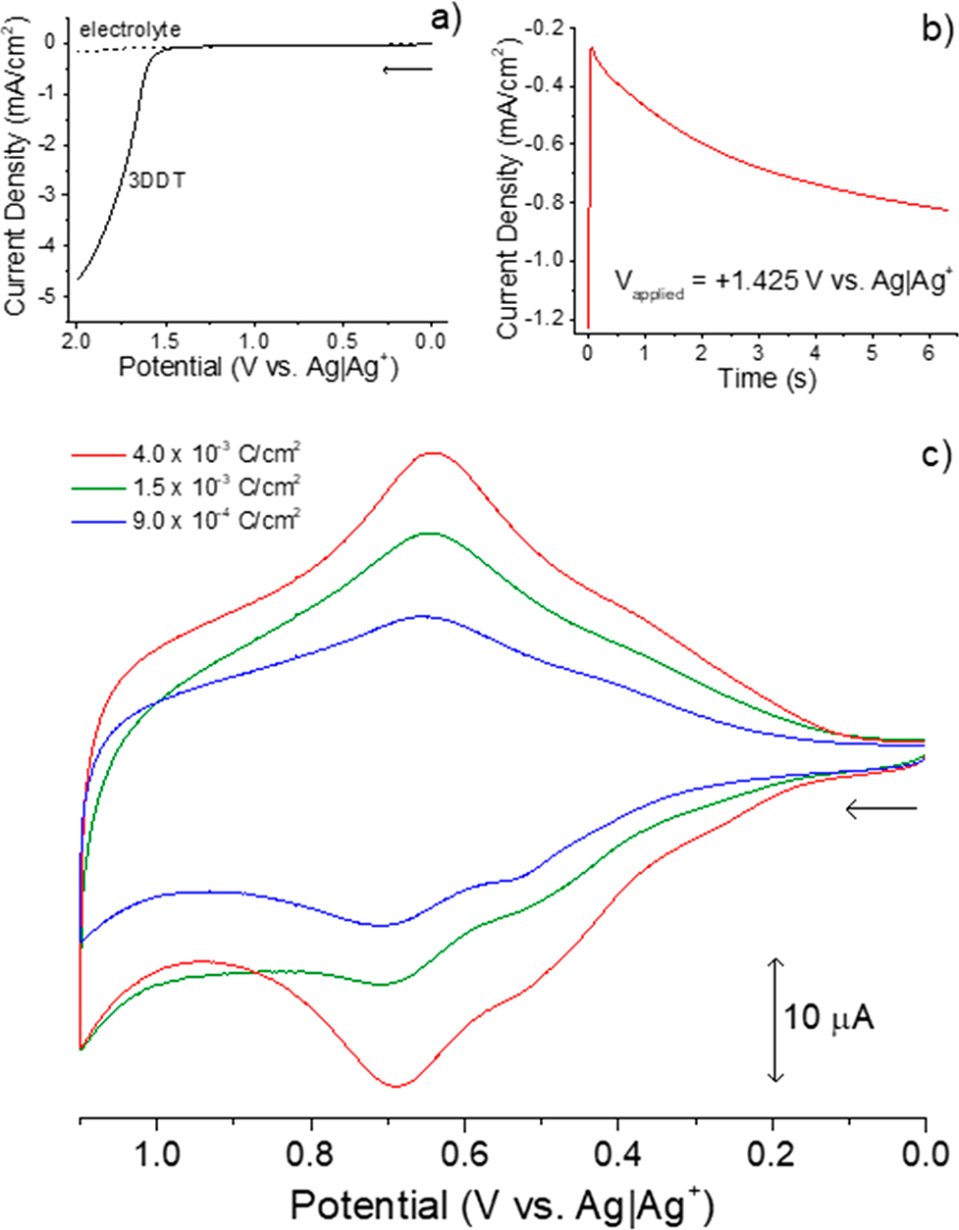
Linear sweep voltammetry (a) of the electrolyte solution (- - -) and the 3-dodecylthiophene monomer (—), ν = 100 mV/s. The electrolyte was tetrabutylammonium hexafluorophosphate (TBAPF_6_, 0.1 M in acetonitrile) while monomer solution was 3-dodecylthiophene (3DDT, 50 mM monomer and 0.1 M TBAPF_6_ in acetonitrile). Representative chronoamperogram (b) acquired during electropolymerization and electrodeposition of a poly(3-dodecylthiophene) (P3DDT) thin film from the 3DDT monomer solution onto an ITO working electrode to form a conducting polymer modified electrode. Cyclic voltammograms (c) of three P3DDT thin films generated by passing 9.0 × 10^–4^ C/cm^2^ (blue), 1.5 × 10^–3^ C/cm^2^ (green), and 4.0 × 10^–3^ C/cm^2^ (red) during chronoamperometric electropolymerization and electrodeposition, ν = 100 mV/s. For all trials, the working electrode was a functionalized planar indium tin oxide thin film on a glass substrate; the electrode area was defined by an O-ring to an area of 0.76 cm^2^. The counter electrode was an ITO substrate oriented parallel to the working electrode, and the reference electrode was Ag|Ag^+^ (10 mM AgNO_3_, 0.1 M TBAPF_6_ in acetonitrile). See Supporting Information Module 1 for additional experimental details. If performing a dry lab, use this figure as part of Module 1.

Chronoamperometric techniques, where the electrochemical cell is held at a potential sufficient for monomer oxidation, are also used to electropolymerize and electrodeposit PAT films. Representative electrochemical data are shown in Figure 2; importantly, the amount of polymer formed is proportional to charge passed during polymerization.

A linear sweep voltammogram used to determine chronoamperometric parameters for (3-dodecylthiophene, 3DDT) is shown in Figure 2a. Faradaic current is observed positive of +1.425 V indicating oxidation of monomers near the working electrode surface. A typical chronoamperogram obtained while depositing poly(3-dodecylthiophene) (P3DDT) is shown in Figure 2b. Features corresponding to polymer nucleation are difficult to discern from the chronoamperogram alone. However, the observed nonlinear growth in the Faradaic current over time is consistent with contemporaneous oxidation of monomers, oxidation of oligomers of varying lengths, and nucleation and growth. Cyclic voltammograms for three P3DDT-modified electrodes formed via chronoamperometry are overlain in Figure 2c. P3DDT electroactivity is evidenced by broad, asymmetric peaks centered at +0.66 V and observed for all three polymer films. The peak magnitudes increase with increasing charge passed during chronoamperometric electropolymerization, indicating increasing amounts of P3DDT. Note that these peak potentials are substantially less positive than those required for electropolymerization, as extended conjugation in polymer chains relative to individual 3DDT monomers reduces the energy needed for oxidation. The peak breadth results from the distribution of polymer chain lengths; electropolymerization is not expected to yield polymer chains with equivalent numbers of monomers because of a combination of factors including nucleation and growth processes occurring simultaneously, diffusion rate differences for monomers and oligomers, solubility differences as a function of the number of bound monomers, and sterics.^26^

### Spectroelectrochemistry of Poly(alkylthiophene)

**Figure 3:**
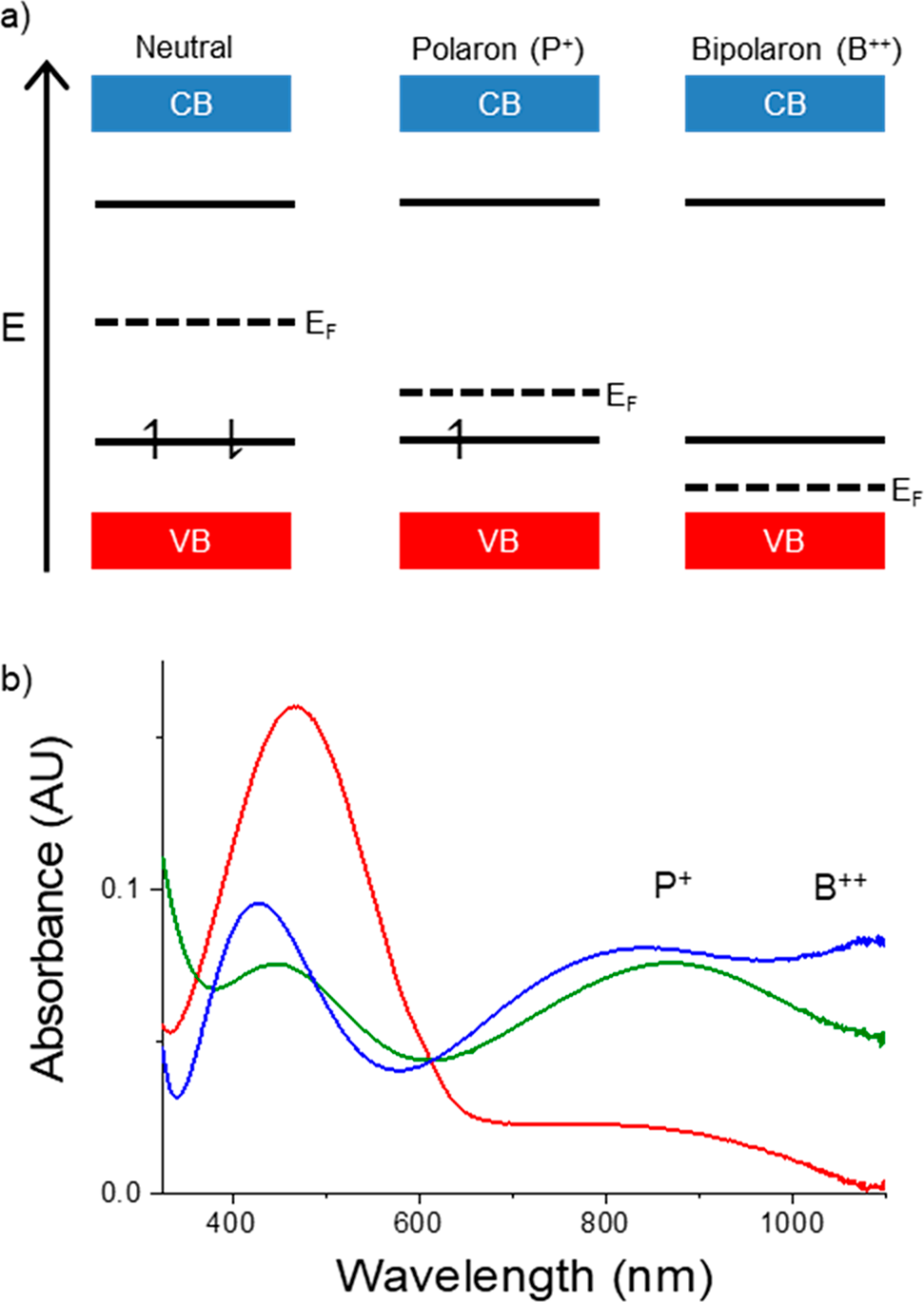
Band diagrams (a) for generic PAT subpopulations and UV–vis absorbance spectra (b) of an electrodeposited P3DDT film that is primarily neutral polymer (red) and when oxidized to form polaronic subpopulations (green) and bipolaronic subpopulations (blue). Prior to spectral analysis, P3DDT was electrodeposited and then the electrochemical cell was poised at potentials sufficient to generate the desired polymer subpopulations. The CPME was removed from the electrochemical cell, rinsed thoroughly, and gently blown dry with a stream of N_2_ gas. Finally, the CPME substrate was taped to a cuvette holder for collection of the UV–vis absorbance spectra. Note that in band diagrams, the implied potential energy axis is in the vertical direction on the page, while the potential axis in electrochemical data is commonly the *x*-axis, positioned horizontally on the page. See Supporting Information Module 3 for additional experimental details. If performing a dry lab, use this figure as part of Module 3.

CPMEs are well-suited to a variety of applications in sensors, lighting, and energy conversion applications, because they undergo potential-dependent optical changes. Oxidized polymer subpopulations, polarons and bipolarons, are spectroscopically distinguishable from neutral polymer subpopulations. To understand spectroscopic differences of polymer subpopulations, consider the band diagrams in Figure 3a for a generic PAT.

The band gap of neutral polymer is the energy difference between the valence band (VB, formed by π-type orbitals in the sp^2^-hybridzed carbons) and the conduction band (CB, formed by corresponding π* orbitals) and typically falls within band gap energies expected for semiconductors. Polymer oxidation yields cations (polarons) and dications (bipolarons), each of which can be delocalized over several monomer units and are electronically shielded by delocalized electrons in neighboring polymer chains. Figure 3b includes absorbance spectra for an electrodeposited P3DDT film that is largely neutral (red) and electrochemically oxidized (green, blue). Neutral P3DDT absorbs near 465 nm, polaronic species absorb near 870 nm, and bipolaronic subpopulations absorb red of 1050 nm. Polarons and bipolarons increase the conductivity of the PAT, analogous to p-type doping of inorganic semiconductors—unoccupied states within the band gap introduce lower-energy electronic transitions.^26^

### Using Solution Redox-Active Species to Characterize a CPME

**Figure 4:**
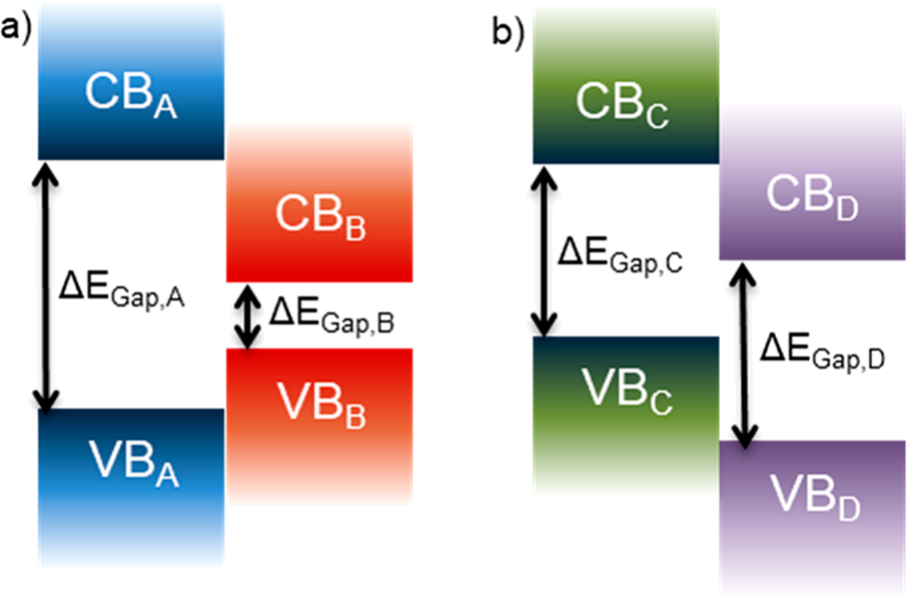
Depiction of the relative energies of the conduction band (CB) and the valence band (VB) of dissimilar materials plotted on a generic potential energy axis. The Type I heterojunction (a) consists of one material with a relatively large bandgap (Δ*E*_Gap,A_) and one material with a relatively small bandgap (Δ*E*_Gap,B_); the smaller bandgap falls within the larger bandgap. In the Type II heterojunction (b), the conduction band energy of one material is lower than that of the second material (CB_D_ < CB_C_); similarly, the valence band energy of one material is lower in potential than that of the second material (VB_D_ < VB_C_).

In organic electronics, multiple dissimilar layers form heterojunctions that separate or allow for recombination of charge carriers. Photogenerated charge carriers are separated at conducting polymer–fullerene interfaces in photovoltaics. CPMEs can serve as charge carrier transport layers in organic light emitting diodes.^4^ The CPME density of states distribution relative to that of the other interfacial material(s) gives rise to the heterojunction functionality, motivating examination of the CPME band electronic structure. Band diagrams for Type I heterojunctions (e.g. light emitting diodes) and Type II heterojunctions (e.g. photovoltaics) are provided in Figure 4. The relative energies of the materials’ bands facilitate the desired charge movement.

**Figure 5:**
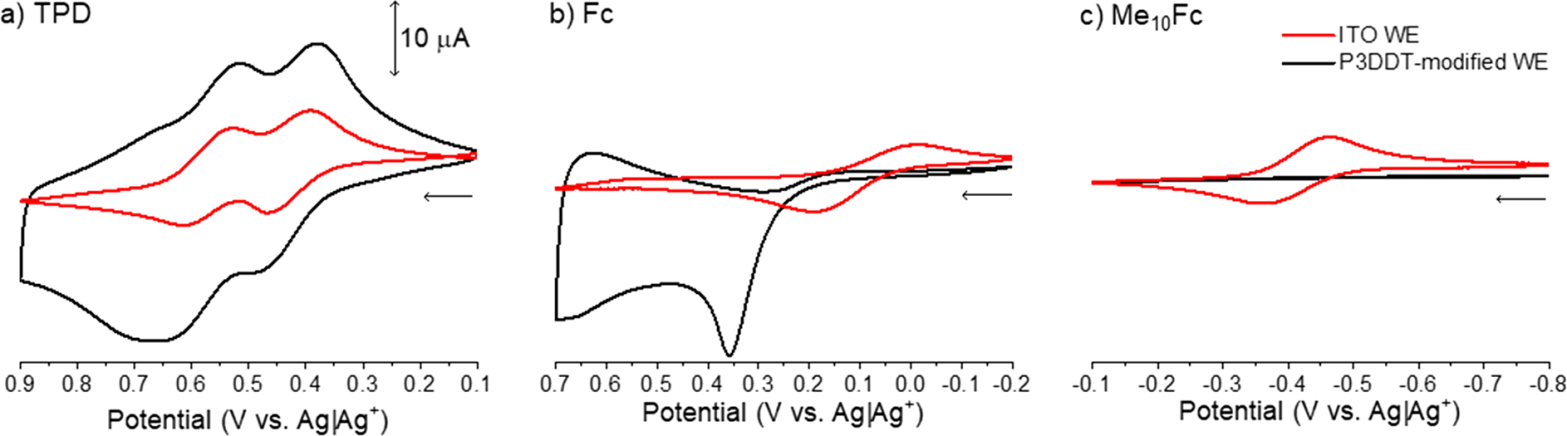
Cyclic voltammograms of TPD (a), ferrocene (Fc, b), and decamethylferrocene (Me_10_Fc, c) on an ITO working electrode (red) and on a P3DDT-modified electrode (black); ν = 50 mV/s, 1 mM redox active species, and 0.1 M TBAPF_6_ in acetonitrile. P3DDT was electrodeposited using chronoamperometry as described previously. See Supporting Information, Module 4 for additional experimental details. If performing a dry lab, use this figure as part of Module 4.

Redox-active small molecules in electrolyte solutions including ferrocene derivatives^27^ and viologen derivatives^43^—molecules with redox potential that can be tuned over 100s of mV using electron-donating or electron-withdrawing substituents—are used to characterize the CPME density of states distributions. The CPME is the working electrode in a three-electrode electrochemical cell. Cyclic voltammetry is used to determine whether electron transfer between the two materials occurs, and cyclic voltammograms of the same redox-active species on a metal electrode (continuous density of states) serve as the control. Figure 5 contains cyclic voltammograms for three redox-active species on an ITO working electrode (red) and a CPME (black). Electrochemically reversible oxidation of all three redox probes is observed on the metal electrode.

When the CPME is the working electrode (Figure 5a–c, black), redox probe electroactivity is observed in the potential windows corresponding to the polaronic and bipolaronic P3DDT. The one-electron oxidation of Me_10_Fc is not observed on P3DDT because there are no polymer states of appropriate energy for electron transfer; the polymer valence band energy is higher in energy than the Me_10_Fc formal potential. Because Me_10_Fc^+^ is not formed, reduction of Me_10_Fc^+^ to Me_10_Fc is not observed on P3DDT. Oxidation of ferrocene is observed on P3DDT, but subsequent reduction is inhibited by lack of P3DDT states negative of 0.0 V. The 2-electron oxidation and subsequent reduction of TPD is amplified relative to the electrochemical activity observed on ITO, as the presence of the electrodeposited P3DDT increases electrode surface area.

## Activity Descriptions

### Module 1: Fabrication of Conducting Polymer Modified Electrodes (CPMEs) Using Electropolymerization and Electrodeposition

In Module 1, researchers electrodeposited P3DDT from monomer solutions onto ITO working electrodes using cyclic voltammetry and chronoamperometry. Integration of voltammetric and amperometric signals reveals relationships between charge passed during a potential sweep or a potential step and the amount of PAT. The CPMEs are chemically and mechanically stable in air for at least one month. Additional skills acquired and/or practiced include solution preparation, assembly of a 3-electrode electrochemical cell, operation of a potentiostat, and interpretation of electrochemical data.

### Module 2: “Watching” Electrodeposition with Atomic Force Microscopy

**Figure 6:**
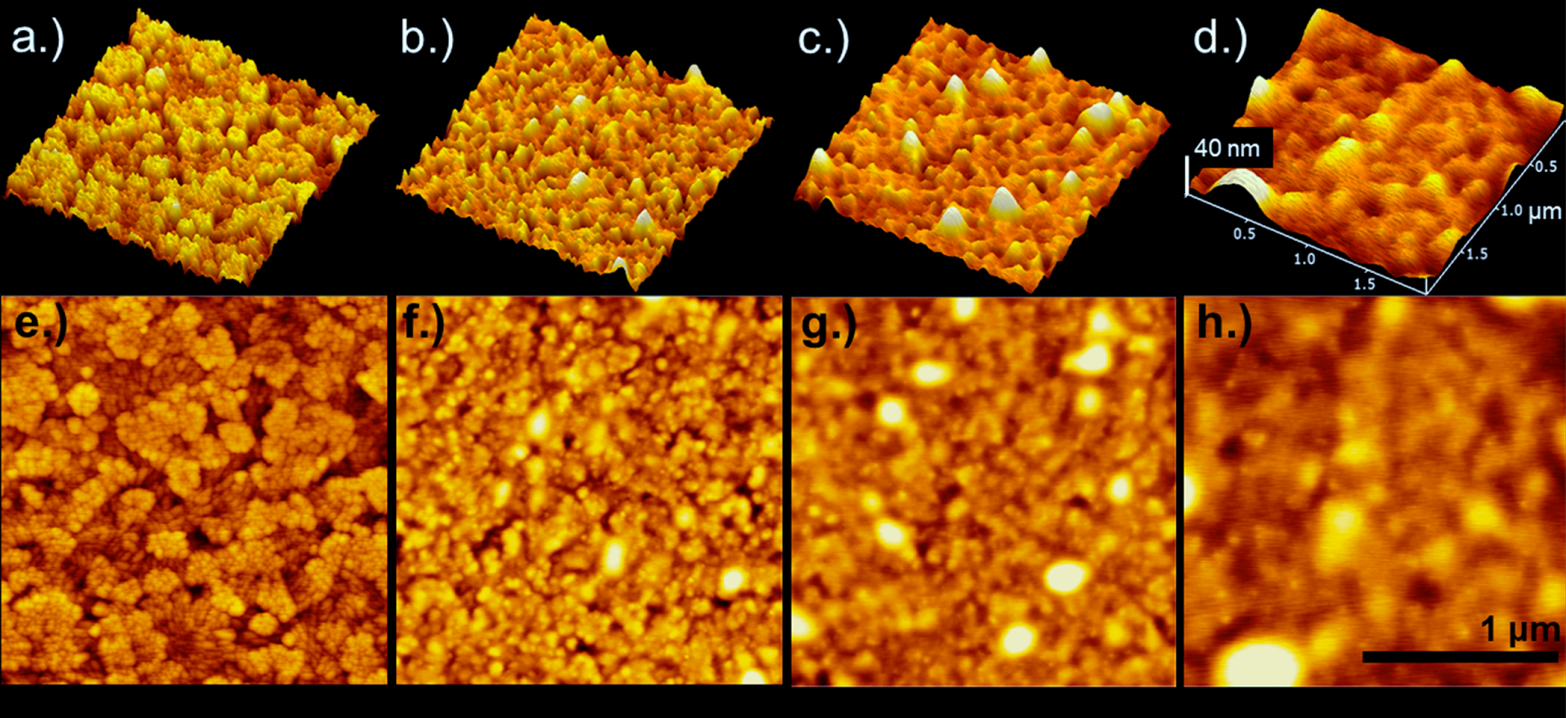
AFM topographical images presented in three dimensions (top) and two dimensions (bottom) of ITO (a, e), P3DDT where the charge passed during electrodeposition was 9.0 × 10^–4^ C/cm^2^ (b, f), 1.5 × 10^–3^ C/cm^2^ (c, g), and 4.0 × 10^–3^ C/cm^2^ (d, h).^44^ The dimensions given in (d) and (h) correspond to (a–d) and (e–h) respectively. See Supporting Information, Modules 1 and 2 for additional experimental details. If performing a dry lab, use this figure as part of Module 2.

To connect observed electrochemical signals to molecular-level processes, researchers visualize polymer electrodeposition by collecting topographical AFM images of PAT on ITO during Module 2. Images corresponding to bare ITO working electrode and three different P3DDT samples are shown in Figure 6.^44^

An AFM image of a bare ITO electrode (Figure 6a, e) enables researchers to distinguish oxide from polymer. PAT nucleation sites are apparent as small, sharp bright spots (*Q* = 9.0 × 10^–4^ C/cm^2^, Figure 6b, f). The areas of the polymeric features increase as the amount of charge passed during electropolymerization increases, which is evidence of PAT growth from nucleation sites. Notably, the height of the polymer features does not increase with charge passed, and instead conformal polymer coverage of the ITO electrode is reached (Figure 6d) yielding a relatively smooth, PAT film. While Module 1 helps students understand electropolymerization at the molecular level, Module 2 provides students with a more macroscopic perspective of ways nucleation and growth yield rough and patchy polymer films. Here 3DDT was chosen rather than more common thiophene monomers with shorter alkyl chains (3-hexylthiophene) because the increased disorder resulting from the longer alkyl chain enabled easier observation of nucleation sites. Module 2 is particularly important for students who will test the impacts of surface modifications on device functionality in organic electronics.

### Module 3: Spectroelectrochemistry of Poly(alkylthiophenes)

**Figure 7:**
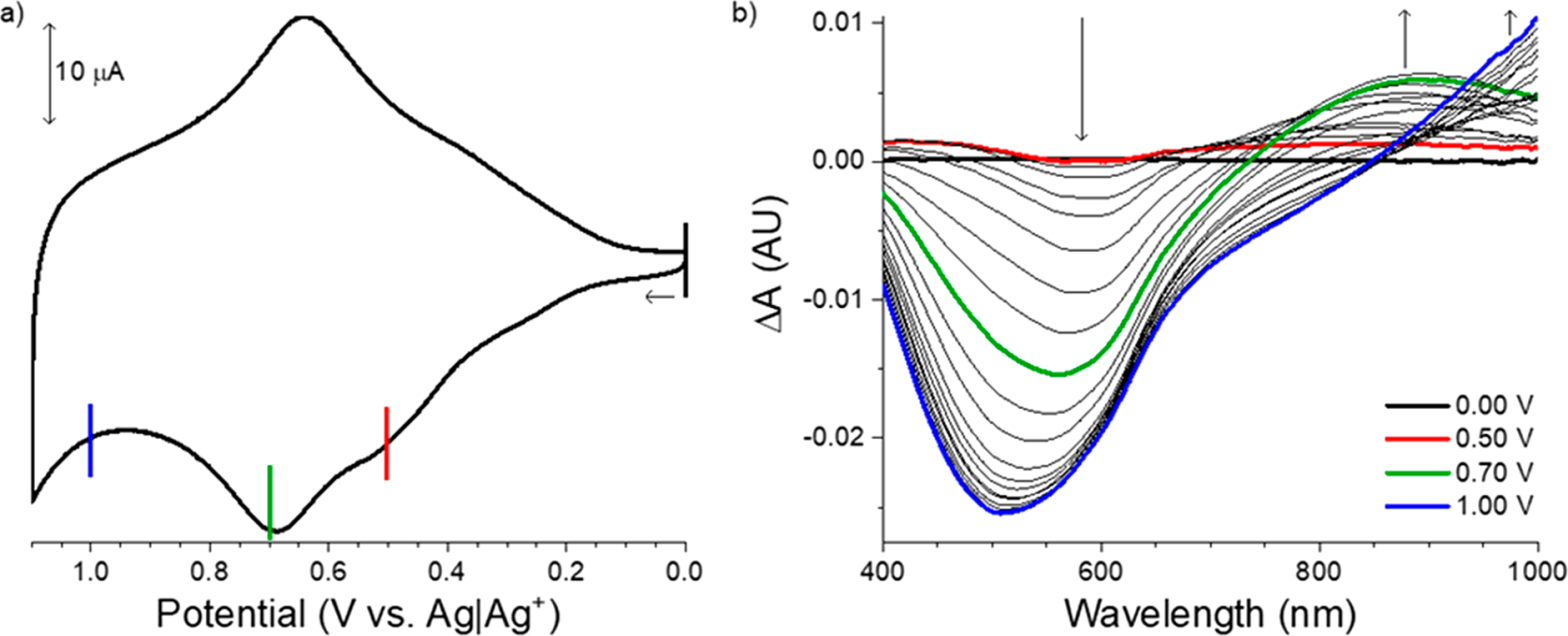
Cyclic voltammogram (a) of an electropolymerized and electrodeposited P3DDT-modified electrode. Difference spectra (b) show the changes in UV–vis absorbance as the P3DDT is oxidized, obtained using a spectroelectrochemical cell placed in the light path of a diode array UV–vis spectrophotometer. The spectral blank was obtained when the cell potential was 0.0 V. The red line on the left indicates the potential at which the red difference spectrum on the right was obtained. Similarly, the green and blue lines (left) show the potentials at which the green and blue difference spectra (right) were obtained. See text for additional discussion and Supporting Information, Module 3, for additional experimental details. If performing a dry lab, use this figure as part of Module 3.

In Module 3, researchers make spectroelectrochemical measurements of electrodeposited P3DDT/ITO interfaces to observe and interpret potential-dependent optical features. By monitoring an electrochemical signal and a spectroscopic signal simultaneously, it is possible to observe polaron and bipolaron formation (for instance). Figure 3b shows the absorbance spectra for neutral, polaronic, and bipolaronic P3DDT, while Figure 7 shows the changes in absorbance that accompany the forward potential sweep, demonstrating the use of spectroelectrochemistry to monitor changes in relative concentrations of polymer subpopulations.

Spectroelectrochemical experiments add specificity to electrochemical or spectroscopic experiments conducted in isolation. By measuring spectral changes as the cell potential is swept positively, the Faradaic current can be correlated with spectral signatures of polymer subpopulations. As the applied potential approaches +0.7 V, a decrease in absorbance corresponding to neutral populations of P3DDT near 465 nm is observed, shown here as a negative change in absorbance in the difference spectra of Figure 7b. Simultaneously a positive change in absorbance or an increase in absorbance is observed near 875 nm, corresponding to the absorbance of P3DDT polarons. Finally, as the potential approaches +1.0 V, decreases in absorbances corresponding to neutral and polaronic P3DDT are observed, while an increase in absorbance corresponding to bipolaronic P3DDT is observed in red of 950 nm. Together, these data demonstrate means of systematically controlling CPME optoelectronic properties by varying relative concentrations of polymer subpopulations with electrochemical oxidation.^26,28^

### Module 4: Analyses of CPMEs Using Solution Redox-Active Species

In Module 4, researchers probe the P3DDT electronic structure using decamethylferrocene, ferrocene, and TPD. Comparison of the cyclic voltammograms of these probe molecules on a bare ITO working electrode to the same cyclic voltammograms collected using a P3DDT-modified ITO electrode demonstrates the impacts of polaron and bipolaron subpopulations on polymer conductivity, as shown in Figure 5. By combining complementary electrochemical and spectroscopic data obtained in Module 3 with redox probe data collected in Module 4, students are equipped to extract some important structure–property relationships in CPMEs.

## Conclusions

These training modules have been used to prepare first-year graduate students for research, as the conceptual content, analytical skills, and interrelated analyses developed through modules built on a typical undergraduate chemistry curriculum. Student researchers fabricate CPMEs and characterize the resulting electrodes with multiple, complementary techniques. In Modules 1 and 2, student researchers learn how to build chemically rational explanations for the electrochemical signals observed during electropolymerization and electrodeposition. In Modules 3 and 4, student researchers measure and explain structure–property relationships in CPMEs. Ultimately, these modules are intentionally designed learning experiences situated in the exciting field of organic electronics.

## Experimental Section

### Materials

Acetonitrile (HPLC grade), 3-dodecylthiophene (3-DDT, 97%), 3-thiopheneacetic acid (3-TAA, 98%), ferrocene (Fc, 99%), decamethylferrocene (Me_10_Fc, 97%), *N*,*N*′-*bis*(3-methylphenyl)-*N*,*N*′-diphenylbenzidine (TPD, 99%), and hydroioidc acid (57% by wt) were purchased from Sigma/Millipore and used as received. Tetrabutylammonium hexafluorophosphate (TBAPF_6_, 99.0%) was purchased from Sigma/Millipore and recrystallized from ethanol. Monomer solutions (50 mM monomer and 0.1 M TBAPF_6_ in acetonitrile) were made fresh daily.

Indium tin oxide (∼100 nm on glass, sheet resistance ∼15 Ω/sq) was purchased from Colorado Concept Coating LLC. Prior to use the ITO substrates were cut into 1-in. squares and cleaned as follows: detergent wash (dilute Triton X-100) and rinse with 18 MΩcm water, 15 min sonication in 18 MΩcm water, rinse with absolute ethanol, and 15 min sonication in absolute ethanol. ITO substrates were stored in absolute ethanol until the following week, just prior to use. ITO working electrodes were functionalized with 3-thioiphene acetic acid using a procedure from literature,^26^ described in detail in the SI. All reagents and solvents are commercially available, and group work plus distributed experimentation can further reduce costs. If the costs of the indium tin oxide coated glass substrates prove to be cost prohibitive, tin oxide coated glass substrates can be used as a substitute.

Electrochemical and spectroelectrochemical experiments were performed in a custom three-electrode spectroelectrochemical cell. The ITO working electrode area was defined by a Viton-O ring (0.76 cm^2^). The counter electrode was a second ITO substrate, and the reference electrode was Ag^+^ (10 mM AgNO_3_, 0.1 M TBAPF_6_ in acetonitrile)|Ag (Bioanalytical systems).

### Hazards

***Caution!** Hydroiodic acid is a strong acid that may be corrosive to metals and causes severe skin burns and eye damage.* All manipulations should be performed on the smallest practical scale and in a fume hood. Waste should be stored in its own appropriately labeled container.

***Caution!** Ferrocene and decamethylferrocene are flammable solids known to cause reproductive toxicity if swallowed or inhaled.* All manipulations should be performed on the smallest practical scale and in a fume hood.

Multiple other hazardous reagents are used during this procedure. Acetonitrile is highly flammable and harmful if swallowed, inhaled, or contacted with skin. Ethanol and 3-dodecylthiophene are highly flammable and irritants. Personal protective equipment including safety glasses and gloves should be worn. All solution preparations should be performed in a fume hood. Waste should be collected in appropriately labeled containers and disposed of in accordance with local regulations.

### Instrumentation

Electrochemical experiments were performed with a CHI 760E bipotentiostat. An Agilent Cary 8454 diode array UV–vis spectrophotometer was used to collect spectra of the polymer thin films. A Hitachi 5100n multifunctional scanning probe microscope was used as an atomic force microscope (AFM), operated in tapping mode using a self-sensing cantilever to collect topographical images of the polymer films. If scanning probe microscopy is not available, use of the images provided in Figure 6 in lieu of performing Module 2 is encouraged. Given the importance of UV–vis spectroscopy in the undergraduate curriculum, Module 3 can be adapted for the instrument available in the local setting with a minimal impact on the module goals (see SI).

## Supporting Information Available

Detailed procedures of the 4 modules written for first-year graduate students, structured documentation for each module, in- and postmodule questions, and rubrics used for assessment of student learning outcomes (PDF) (DOCX)
