# Supplementary material for: Understanding Polymer Electrodeposition and Conducting Polymer Modified Electrodes Using Electrochemistry, Spectroscopy, and Scanning Probe Microscopy
Source: J Chem Educ. 2023 Sep 13;100(10):4062–71. doi: 10.1021/acs.jchemed.3c00656 (PMC10571039; doi:10.1021/acs.jchemed.3c00656)
Supplement: Supplementary file 2 — ed3c00656_si_002.docx [file ed3c00656_si_002.docx]

**Supporting information**

**Understanding polymer electrodeposition and conducting polymer modified electrodes using electrochemistry, spectroscopy, and scanning probe microscopy**

Jessica M. Bone, Judith L. Jenkins*

Department of Chemistry, Eastern Kentucky University, Richmond, KY 40475

*Corresponding Author

Email: [judy.jenkins@eku.edu](mailto:judy.jenkins@eku.edu)

**Table of Contents**

p2 Introduction

p2 – 3 Safety, Hazards, Materials, Equipment

p3 Suggested Literature Resources; ITO electrode preparation procedures

p4 Figure SI1. Electrochemical and spectroelectrochemical cell design

p5 – 9 Module 1**:** Fabrication of Conducting Polymer Modified Electrodes via Electropolymerization and Electrodeposition

p10 – 11 Module 2: “Watching” electrodeposition with atomic force microscopy

p12 – 15 Module 3: Spectroelectrochemistry of poly(alkylthiophene)

p16 – 19 Module 4: Analysis of CPMEs using solution redox-active species

p20 - 22 Rubrics for assessment of learning outcomes

p23 References

**Introduction**

In these modules you will observe electropolymerization and polymer electrodeposition using a combination of electrochemistry, spectroscopy, and atomic force microscopy topographic images. Together, these modules are designed to build practical skills, to deepen fundamental chemical understanding, and to develop researchers who will contribute to future advances with conducting polymers and conducting polymer-modified electrodes.

To get the most out of the modules, answer the questions embedded in procedure while performing the experiments. These questions provide structure for observations and draw attention to important features in the data. Additionally, the embedded questions facilitate preliminary analysis, and help develop chemical thinking skills during interpretation. More detailed data analysis will be completed through the questions at the end of the module.

These procedures work well for the 3-dodecylthiophene monomer (3DDT). If using a different alkylthiophene monomer, modifications of monomer concentrations and deposition charges may be required.

**Hazards**

Caution! Hydroiodic acid is a strong acid which may be corrosive to metals and causes severe skin burns and eye damage. All manipulations should be performed on the smallest practical scale and in a fume hood. Waste should be stored in its own appropriately labeled container. Caution! Ferrocene and decamethyl ferrocene are flammable solids known to cause reproductive toxicity if swallowed or inhaled. All manipulations should be performed on the smallest practical scale and in a fume hood. Multiple other hazardous reagents are used during this procedure. Acetonitrile is highly flammable and harmful if swallowed, inhaled or contacted with skin. Ethanol and 3-dodecylthiophene are highly flammable and irritants. Personal protective equipment including safety glasses and gloves, should be worn. All solution preparation should be performed in a fume hood. Waste should be collected in appropriately labeled containers and disposed of accordance with local regulations.

**Materials**

*ITO cleaning and functionalization* - hydroiodic acid (57% by wt), 18 MΩ·cm water, ethanol (200 proof), Triton X-100, 3-thiophene acetic acid (3-TAA); ITO thin films – here, ITO (~100 nm, sheet resistance 15 Ω/sq on glass) was purchased from Colorado Coating Concept LLC and cut into 1-inch squares prior to the preparation procedure given in the following section.

*Electrochemistry*

- Monomer: 3-dodecylthiophene (3DDT)
- Electrolyte: tetrabutylammonium hexafluorophosphate (TBAPF_6_)
- Solvent: acetonitrile
- Redox probes: ferrocene (Fc), decamethylferrocene (Me_10_Fc), *N*,*N*’-*bis*(3-methylphenyl)-*N*,*N*’-diphenylbenzidine (TPD)

**Equipment**

*Electrochemistry*

- Potentiostat with accompanying electrochemical leads, computer with software for instrument control
- Reference electrode (RE) Ag|Ag^+^ (10 mM AgNO_3_, 0.1 M TBAPF_6_ in acetonitrile); ITO counter electrode (CE); ITO working electrode (WE); functionalized ITO working electrode (WE)*
- 3-electrode electrochemical cell in which the WE and CE can be physically parallel to one another such as the custom sandwich cell shown in Figure SI1 or a microscope slide holder. A portion of Module 2 relies on an electrochemical cell that can be placed in the beam path of a UV-Vis absorbance spectrophotometer. However, this portion of the module can be omitted in the absence of an appropriate cell.

* Module 3 relies on optically transparent working electrodes. Module 2 relies on a WE that serves as a substrate for AFM measurements. If only performing Modules 1 and/or 4, electrodes other than ITO on glass substrates could be used for the CE and WE.

*Spectroscopy*

- A UV-Vis absorbance spectrophotometer capable of probing at least 400 nm – 900 nm will be sufficient for all experiments described. With the exception of Module 3, Part B Tape will be used to affix polymer-modified ITO electrodes to the cuvette holder such that the polymer is in the beam path of the spectrophotometer.
- Module 3, Part B requires that the electrochemical cell be placed directly into the beam path of the spectrophotometer, which will likely require removal of the cuvette holder. Additionally, this portion of the module is best completed with a diode array spectrophotometer.

*Atomic Force Microscopy*

- A scanning probe microscope with atomic force microscopy (AFM) capabilities will be needed from completion of Module 2. We recommend collecting topographic images in tapping mode with the following additional parameters: 5 μm x 5 μm topography image 4096 lines with 4096 pixels/line.

**Suggested Literature Resources**

For a detailed discussion of thiophene electropolymerization and electrodeposition, see recent works by Heinze^1-2^ (electropolymerization and electrodeposition), Dempsey^3^ (basics of echem and cyclic voltammetry), Jenkins^4^ (for spectroscopy, electrochemistry, and density states in a specific polymer system), and Luscombe^5^ (conducting polymers).

**ITO Electrode Preparation Procedures**

For all ITO electrodes, cut ITO substrates into 1-inch squares and clean using a detergent wash (diluted Triton X-100) and rinse with 18 MΩ·cm water followed by sonication (15 minutes, 18 MΩ·cm water). Next, rinse all squares with absolute ethanol followed by sonication (15 minutes, absolute ethanol). Store the ITO substrates in absolute ethanol until just prior to use. We recommend cutting and cleaning at least thirty 1-inch ITO electrode squares to serve as the CEs and WEs in these modules, some of which will also be functionalized prior to use. Immediately before use or functionalization, dry an ITO square using a stream of air or gaseous nitrogen. Identify the ITO side of the square using a multimeter.

To functionalize the ITO WE, clean and dry the ITO squares using the above procedure. Then, cover the ITO surface with hydroiodic acid (HI, 57% by wt) for 8-10 seconds. Rinse thoroughly (18 MΩ·cm water), dry the square, and then immerse the square in a 3-thiophene acetic acid solution (3-TAA, 100 mM in absolute ethanol) for a minimum of 12 hours and a maximum of 48 hours. Just prior to use, remove the ITO square from the 3-TAA solution, rinse thoroughly with absolute ethanol, and dry using a stream of air or gaseous nitrogen. Identify the ITO side of the square using a multimeter, assemble the electrochemical cell, and promptly fill the cell with the desired solution.

Figure SI1. Electrodeposited P3DDT thin film on ITO (a). CPME taped to cuvette holder (b, c) to enable measurements collected in Module 3. Sandwich electrochemical cell (d) used for all electrochemical and spectroelectrochemical work describe in the manuscript and in Modules 1-4. Drawings for machining sandwich electrochemical cell (e). Alternatively, use a microscope slid holder as the electrochemical cell. Place 2 planar electrodes (WE and CE) parallel to one another in slots; insert RE between WE and CE.

**Module 1: Fabrication of Conducting Polymer Modified Electrodes (CPMEs) Electropolymerization and Electrodeposition**

In this module, you will use two different electropolymerization techniques – cyclic voltammetry and chronoamperometry – to electrodeposit poly(3-dodecylthiophene) onto indium tin oxide (ITO) working electrodes.

Use Tables 1 and 2 to record quantitative observations during data collection. Think through and briefly answer the questions embedded in the procedure *during* data collection. Use the questions at the end of the module for more thorough data processing and analysis.

**Learning Outcomes**

*After completion of this module, you will be able to…*

- Electropolymerize and electrodeposit PAT using both cyclic voltammetry and chronoamperometry, and obtain cyclic voltammograms of the resulting PAT thin films
- Qualitatively explain the voltammograms or amperograms obtained during electrodeposition using particle representations
- Quantitative relate the charge passed during the chronoamperometric deposition to amount of electroactive PAT on the CPME

Table 1 – For Module 1, Part A

| Oxidative Sweep | Oxidation peak potential, *E_p_* (V vs. Ag\|Ag^+^) | Oxidation peak current, *i_p_* (A) |
| --- | --- | --- |
| 1 |  |  |
| 5 |  |  |
| 10 |  |  |

Table 1, For Module 1, Part B

| Charge density (C/cm^2^) | *E_p_*_, ox_ (V) | *E_p_*_, red_ (V) | *i_p_*_, ox_ (A) | *i_p_*_, red_ (A) |
| --- | --- | --- | --- | --- |
| -4.0 x 10^-3^ |  |  |  |  |
| -1.5 x 10^-3^ |  |  |  |  |
| -9.0 x 10^-4^ |  |  |  |  |

**Module 1: Fabrication of CPMEs via Electropolymerization and Electrodeposition, continued**

**Procedure***

- Prepare 4 ITO CEs, 1 ITO WE, and 3 functionalized ITO WEs
- Prepare 50 mL of the electrolyte solution: 0.1 M TBAPF_6_ (acetonitrile)
- Prepare 25 mL of the monomer solution using some of the prepared electrolyte solution to yield a solution that is 0.05 M 3DDT and 0.1 M TBAPF_6_ in acetonitrile

Solution volumes assume that the assembled electrochemical cell requires 5 mL; if using a cell requiring a different solution volume, adjust the above volumes accordingly.

*All potentials are with respect to an Ag|Ag^+^ nonaqueous RE (10 mM AgNO_3_, 0.1 M TBAPF_6_ in acetonitrile).

Part A: Electropolymerization and electrodeposition of P3DDT with cyclic voltammetry

1. Assemble the electrochemical cell with a fresh ITO CE, an ITO WE (non-functionalized), and the RE. Fill the cell with monomer solution.
2. Cycle the potential between 0.0 V and +1.425 V vs. Ag|Ag^+^ for a total of 10 cycles at a scan rate of 100 mV/s to electropolymerize and electrodeposit P3DDT. Record oxidation peak potentials and currents as a function of cycle number using Table 1.

*Describe one or two of the biggest changes you observe in the voltammogram from the first cycle to the tenth cycle.*

*What features in your voltammogram provide evidence in support of the electropolymerization and electrodeposition mechanisms described by Heinze et. al?^1-2^ Explain.*

1. Taking care not to disturb the WE, remove the monomer solution using a pipette, and refill the cell with electrolyte solution.
2. Record a cyclic voltammogram using the following parameters: potential range 0.0 V to +1.0 V vs. Ag|Ag^+^, 2 cycles, scan rate = 100 mV/s

*Compare the cv of the P3DDT to the cv of ferrocene shown here and note any differences in peak splitting and peak shapes. As part of the comparison, write a half reaction corresponding to the electron transfer reaction occurring in P3DDT as the potential is swept from 0.0 V to +1.0 V and as the potential is swept from +1.0 V to 0.0 V.*

*How might the P3DDT cyclic voltammogram change (if at all) if 20 cycles had been used during electropolymerization and electrodeposition rather than 10 cycles? Explain.*

**Module 1: Fabrication of CPMEs via Electropolymerization and Electrodeposition, continued**

Part B: Electropolymerization and electrodeposition with chronoamperometry

1. Assemble the electrochemical cell with fresh ITO CE, a 3-TAA functionalized ITO WE, and the RE. Fill the cell with monomer solution.
2. To electrodeposit P3DDT, step the potential to +1.425 V vs Ag|Ag^+^ until a charge density of **-4 x 10^-3^ C/cm^2^** is reached.

*Depict any chemical reactions occurring at the working electrode involving 3DDT and P3DDT during this potential step.*

*Why does P3DDT deposit onto the WE instead of diffusing away from the electrode?*

1. Taking care not to disturb the WE, remove the monomer solution using a pipette, and refill the cell with electrolyte solution.
2. Record a cyclic voltammogram using the following parameters: potential range 0.0 V to +1.0 V vs. Ag|Ag^+^, 2 cycles, scan rate = 100 mV/s
3. Disassemble the electrochemical cell, thoroughly rinse the P3DDT with acetonitrile, blow dry with a stream of N_2_, and save in a labeled container, taking care to protect the P3DDT film from dust and scratches. This P3DDT film is stable in air and can be used to complete portions of Modules 2 and 3.
4. Repeat steps 1-5 using a fresh functionalized ITO WE and a fresh ITO CE, stepping the potential to +1.425 V until a charge density of **-1.5 x 10^-3^ C/cm^2^** is reached.

*Before collecting a cyclic voltammogram, predict how the cv of this P3DDT will compare to the P3DDT cv of the -4 x 10^-3^ C/cm^2^ P3DDT sample. Provide a brief rationale for your prediction.*

1. Repeat steps 1-5 sequence using a fresh functionalized ITO WE and a fresh ITO CE, stepping the potential to +1.425 V until a charge density of until a charge of **-9 x 10^-4^ C/cm^2^** is reached.

*Overlay the cv’s for the 3 polymer films deposited using chronoamperometry. Record peak currents and peak potentials as a function of charge passed during electrodeposition.*

*Describe the ways the P3DDT voltammograms change as the charge density passed during electrodeposition increases.*

**Module 1: Fabrication of CPMEs via Electropolymerization and Electrodeposition, continued**

Questions for data processing and analysis

*Peak magnitudes (and peak areas) in cyclic voltammograms are proportional to the concentration of the electroactive species. Using the amperograms and the cv’s collected during Part B, describe the relationship between the charge passed during electrodeposition and the amount of P3DDT polymerized.*

*Assuming non-Faradaic contributions to current are negligible (technically not true, but close enough for now), quantify the amount of P3DDT electrodeposited using each amperogram collected in Part B by converting the charge passed to the moles of 3DDT monomers oxidized.*

*Again, assuming non-Faradaic contributions to current are negligible, quantify the amount of P3DDT electrodeposited by integrating the current passed during an oxidative sweep in a cv for each P3DDT sample from Part B.*

*How do these values compare to one another? Are there advantages to using one type of data over another when quantifying the amount of polymer electrodeposited? Explain.*

*
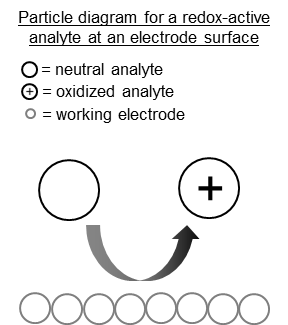
*

*Visualize the 3-TAA functionalized WE at the molecular level as electrodeposition of P3DDT occurs. Use the boxes below to depict the ITO/3-TAA/P3DDT interfaces in two dimensions as a function of time passed during the potential step using a particle diagram (monomers represented as circles; solvent molecules may be omitted).*

*
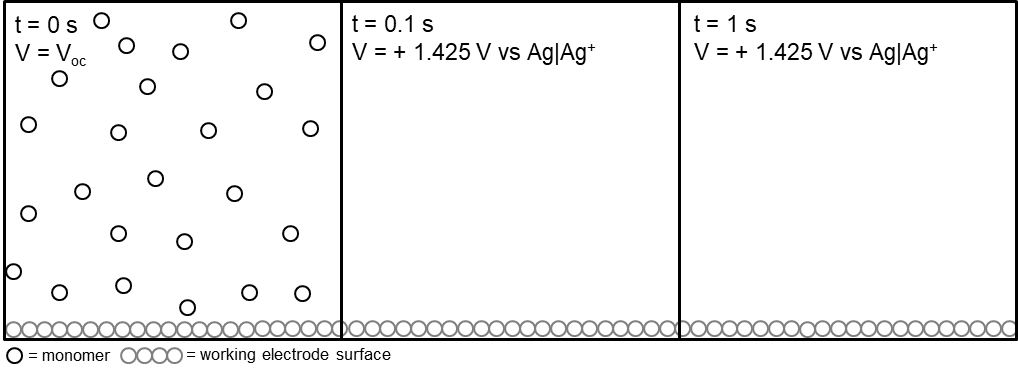
*

**Module 1: Fabrication of CPMEs via Electropolymerization and Electrodeposition, continued**

*Consider electropolymerization and electrodeposition via cyclic voltammetry and via potential step. Is one technique better suited to an application than another? Explain.*

*Can we learn anything about the P3DDT morphology on the working electrode surface from the P3DDT cv’s? For instance, could we distinguish between a single long P3DDT chain and a brush-like P3DDT thin film as depicted here?*

*
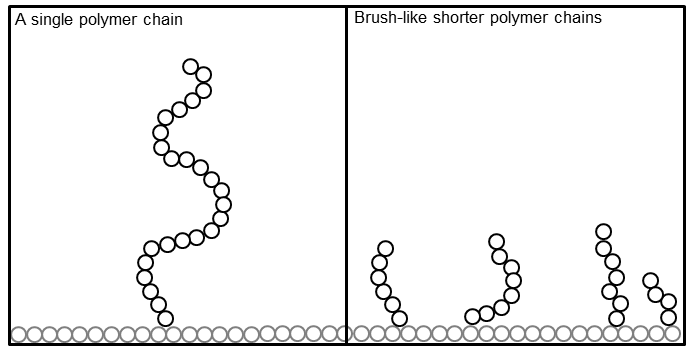
*

*Compare polymer electrodeposition to other fabrication techniques like spin coating or ink-jet printing. Discuss some of the relative advantages and disadvantages of the techniques.*

Note: For comparison, representative data obtained during Module 1 Part A are given in Figure 1 of the manuscript, while the same data for Module 1 Part B are given in Figure 2 of the manuscript.

**Module 2: “Watching” polymer electrodeposition with atomic force microscopy**

In this module, you will collect AFM topographical images of an ITO WE and of the three P3DDT samples electrodeposited in Module 1. Each P3DDT sample provides a snapshot of the electrodeposition. By collecting topographical images of the sample with the least polymer (9 x 10^-4^ C/cm^2^), we are able to see polymer growing from the initial nucleation sites on the ITO surface. When imaging the samples with more polymer, we are able to watch electropolymerization and electrodeposition proceed until the P3DDT conformally covers the ITO.

As you complete this module, consider ways the topographical AFM images compliment the electrochemical characterization of P3DDT. What do we learn from these AFM images that cannot be gleaned from cyclic voltammetry alone?

**Learning Outcomes**

*After completion of this module, you will be able to…*

- Describe the morphology of the electrodeposited PAT on the electrode surface
- Relate changes in observed polymer morphology to differences in electrodeposition parameters
- Connect electrochemical data and topographical images to construct molecular-level understanding of polymer nucleation and growth processes from evidence.

**Procedure**

This module can be completed using the three P3DDT samples electrodeposited in Module 1, Part B. Alternatively, prepare three different P3DDT films on ITO electrodes according to the procedure described in Module 1, Part B. Additionally, prepare one clean, dry ITO electrode using the ITO Preparation Procedure.

When collecting the AFM images, we recommend starting with the clean ITO electrode (no polymer) as a control before imaging the P3DDT samples. It is common to pick up polymer on the AFM tip when imaging the sample with the most polymer (-4.0 x 10^-3^ C/cm^2^), so we recommend collecting any images of that sample last. Ensure all polymer samples are dried with a stream of inert gas before AFM analysis.

1. Collect at least one 5 μm x 5 μm topography image of each electrode following these general steps.* steps.

**Please note that the operating procedures for scanning probe microscopes vary greatly. The general steps are described below, but individuals should take care to modify these steps as needed for a given microscope.*

Mount each electrode on a substrate compatible with the scanning probe microscope you will use.

Place the sample on the microscope, and use the optical microscope to locate the sample.

Mount the AFM tip.

Select the topography image parameters. We recommend collecting at least one 5 μm x 5 μm topography image for each electrode using relatively high resolution. For example, 4096 lines 4096 pixels/line works well. Acceptable AFM topographical images can be acquired using contact mode or tapping mode (dynamic force microscopy).

Align, tune, and engage the AFM tip. Proceed with image collection. Ensure the raw data is saved after data collection. Examine the collected image.

**Module 2: “Watching” polymer electrodeposition with atomic force microscopy, continued**

*Do you see any evidence of tip artifacts? Of noise? Take appropriate steps to mitigate any observed tip artifacts or noise before proceeding to the next image.*

*How would you describe the ITO electrode surface at the nm scale? Is this similar to what you would have expected from visual inspection of the ITO? If different, how so?*

1. Disengage the tip, and move to a different portion of the sample to collect a second image.
2. Repeat the image collection steps. We recommend collecting 3 images examine the uniformity of the ITO (or the lack thereof).
3. After an image is collected, flatten the image and correct for tilt. Then, identify a representative 2 μm x 2 μm square for each sample. Adjust all images to display using the same scale in the z-direction, and compare.

*Before collecting any images of the P3DDT, describe what you expect these polymer samples to look like at nm scale relative to ITO. How might these samples differ in appearance as a function of the charge passed during electropolymerization and electrodeposition?*

1. Repeat steps 1-4 to collect and process images of all three P3DDT samples.
2. After all images have been collected, save the P3DDT samples for use in Module 3.

Questions for data processing and analysis

*Use the AFM images of the ITO electrode to distinguish between areas of ITO and P3DDT on a given P3DDT sample. Examine the polymeric features of the P3DDT sample. Consider the following questions meant to be answered qualitatively.*

- *Do you notice any changes to the polymeric features as a function of charge passed during electrodeposition? Explain.*
- *Are all P3DDT samples conformally covered with polymer, or are patches of ITO visible between polymer? If ITO is visible, does the amount of ITO visible change with charge passed during P3DDT deposition? Are any trends observable?*

*Use the AFM software imaging software to more quantitatively examine the samples. Pay particular attention to the heights of the polymeric features, the surface roughness, and the relative sizes of ITO and polymer patches. Quantitatively compare and contrast the images.*

*Return to the particle diagrams generated during Module 1 – How would the single chain or brush particle depictions of the P3DDT need to be modified to more accurately incorporate the AFM data?*

*What features in your AFM images provide evidence in support of polymer nucleation and growth? Explain.*

Note: For comparison, representative data obtained during Module 2 are given in Figure 6 of the manuscript.

**Module 3: Spectroelectrochemistry of poly(3-dodecylthiophene)**

During Part A of this module, you will use electrochemical oxidative doping to vary the relative concentrations of neutral, polaronic, and bipolaronic P3DDT. You will collect UV-Vis absorbance spectra of the P3DDT as a function of oxidative doping, relating the polymer electrochemical and optical properties to one another.

**Learning Outcomes**

*After completion of this module, you will be able to…*

- Perform spectroelectrochemistry by collecting UV-Vis absorbance spectra during electropolymerization and electrodeposition
- Spectroscopically identify and distinguish neutral PAT, polaronic PAT, and bipolaronic PAT subpopulations
- Use spectroscopic and spectroelectrochemical data to rationalize why and/or predict how optoelectronic properties of the CPME change when the relative concentrations of neutral, polaronic, and bipolaronic PAT change.

Use Tables 3 and 4 to record observations during and after data collection. Think through and briefly answer the questions embedded in the procedure during data collection. Use the questions at the end of the module for more thorough data processing and analysis.

Table 3 – For Module 3, Part A

Record the absorbance values for the electronic transition characteristic of the neutral, polaronic, and bipolaronic P3DDT (500 nm, 850 nm, and 1000 nm respectively) for each spectrum collected in Part A.

| Oxidative Potential (V vs. Ag\|Ag^+^) | Absorbance at 500 nm (AU) | Absorbance at 850 nm (AU) | Absorbance at 1000 nm (AU) |
| --- | --- | --- | --- |
| 0.0 |  |  |  |
| 0.6 |  |  |  |
| 1.0 |  |  |  |

Table 4, For Module 3, Part B

Observe the changes in the absorbance spectra of P3DDT as the potential is scanned positively. For each potential range, indicate whether the absorbance increased, decreased, or stayed the same.

| Potential Range (vs. Ag\|Ag^+^) | Absorbance at  500 nm (ΔA) | Absorbance at  850 nm (ΔA) | Absorbance at  1000 nm (ΔA) |
| --- | --- | --- | --- |
| 0.0 V 🡪 +0.5 V | *Increased, decreased, or no change?* |  |  |
| +0.5 V 🡪 +0.7 V |  |  |  |
| +0.7 V 🡪 +1.0 V |  |  |  |
| +1.0 V 🡪 +0.7 V |  |  |  |
| +0.7 V 🡪 +0.5 V |  |  |  |
| +0.5 V 🡪 0.0 V |  |  |  |

**Module 3: Spectroelectrochemistry of poly(3-dodecylthiophene), continued**

**Procedure***

- This module can be completed using one of the polymer films electrodeposited in Part B of Module 1. Alternative, electrodeposit one P3DDT sample on a functionalized ITO WE using the procedure described in Module 1, Part B.
- Prepare 25 mL of the electrolyte solution: 0.1 M TBAPF_6_ (acetonitrile) and one ITO CE
- Dry one clean ITO square to use as the blank when collecting UV-Vis absorbance spectra.

*All potentials are with respect to an Ag|Ag^+^ nonaqueous RE (10 mM AgNO_3_, 0.1 M TBAPF_6_ in acetonitrile).

Part A: Potential Dependent Spectra

1. Assemble the electrochemical cell with a fresh ITO CE, the P3DDT WE, and the RE. Fill the cell with electrolyte solution.
2. Record a cyclic voltammogram using the following parameters: potential range 0.0 V to +1.0 V vs. Ag|Ag^+^, 2 cycles, scan rate = 100 mV/s

*Look closely – How many features are seen in the oxidative sweep of the cv? At what potentials?*

1. Step the potential to 0.0 V vs. Ag|Ag^+^ for 60 seconds. Promptly disconnect the electrochemical leads, remove the P3DDT WE, rinse the P3DDT with acetonitrile, and dry with a stream of gaseous nitrogen.
2. Using a clean non-functionalized ITO slide as the blank, collect an absorbance spectrum of the P3DDT from 400 nm to at least 1000 nm. Record your observations in Table 2. Ensure the ITO substrate is attached to the spectrophotometer sample holder such that the P3DDT covers the beam path as shown here.


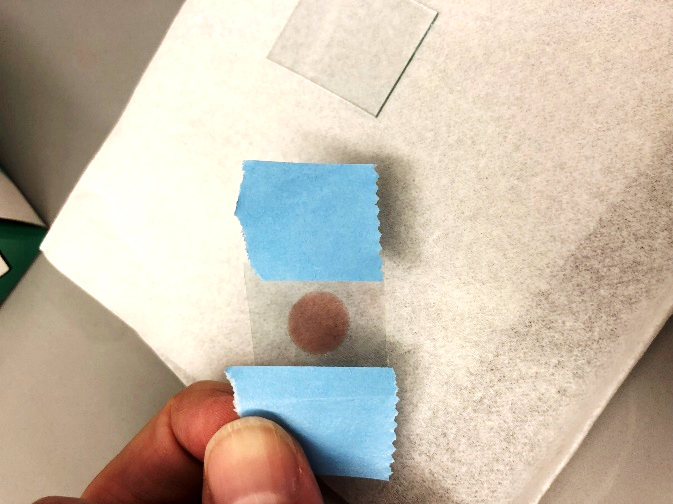

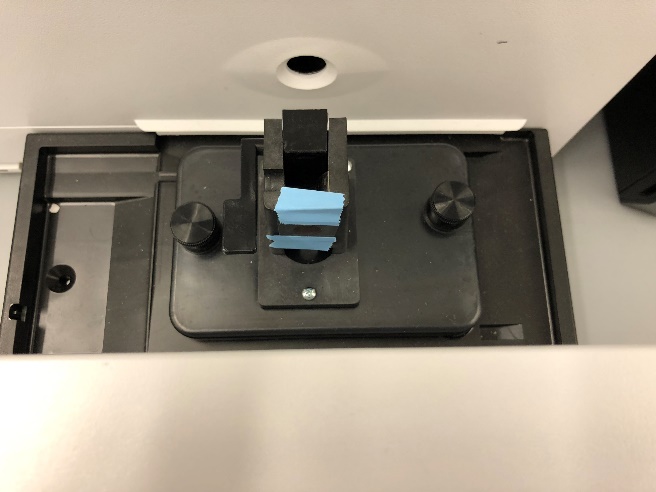

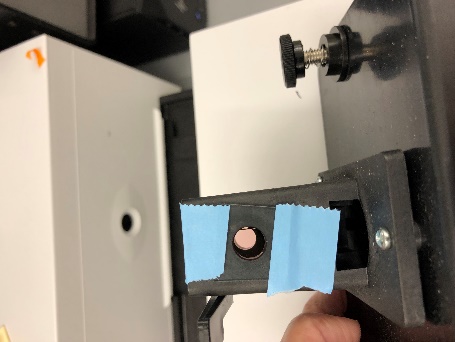


P3DDT

1. Return the P3DDT WE to the electrochemical cell and fill the cell with electrolyte solution.
2. Step the potential to +0.6 V vs. Ag|Ag^+^ for 60 seconds. Promptly disconnect the electrochemical leads, remove the P3DDT WE, rinse with acetonitrile, and dry with a stream of gaseous nitrogen.

*Predict – how might the absorbance spectrum change after the P3DDT was poised at* +0.6 V Ag|Ag^+^ for 60 seconds*?*

1. Collect an absorbance spectrum of the P3DDT WE using the same parameters used in step 4.

*Compare – did the absorbance spectrum change as you predicted?*

1. Return the P3DDT WE to the electrochemical cell and fill the cell with electrolyte solution.
2. Step the potential to +1.0 V vs. Ag|Ag^+^ for 60 seconds. Promptly disconnect the electrochemical leads, remove the P3DDT-functionalized ITO WE, rinse with acetonitrile, and dry with a stream of gaseous nitrogen.
3. Collect an absorbance spectrum of the P3DDT WE using the same parameters used in step 4.

*Suggest one or more molecular-level explanation for the variations in P3DDT optical properties as a function of oxidation potential.*

**Module 3: Spectroelectrochemistry of poly(3-dodecylthiophene), continued**

Part B: Spectroelectrochemistry

Here, you will record a cyclic voltammogram and absorbance spectra simultaneously. This allows us to monitor the potential-dependent optical properties of P3DDT without removing the polymer from the electrochemical cell. This portion of the module is most easily performed with a diode array spectrophotometer. *If you choose not complete Part B, proceed to the questions at the end of this module.*

1. Assemble the electrochemical cell with a fresh ITO CE, the P3DDT WE, and the RE. Fill the cell with electrolyte solution.
2. Place the electrochemical cell in the spectrophotometer beam path and connect the electrochemical leads. Ensure that the cell will remain in place for several minutes.
3. Collect the spectroscopic blank with the P3DDT (in the beam path) poised at 0.0 V vs. Ag|Ag^+^. We recommend a 1-second spectral acquisition time. Because the blank is the neutral P3DDT, the subsequent spectra will be difference spectra, highlighting the *changes* in the polymer optical properties as a function of electrochemical oxidation.
4. Collect a cyclic voltammogram and absorbance spectra simultaneously by following these steps.

- Adjust the potentiostat to record a cyclic voltammogram with the potential range 0.0 V to +1.0 V vs. Ag|Ag^+^, 1 cycle, scan rate = 20 mV/s, and a quiet time of 5 seconds. *Do not start the electrochemical measurement.*
- By altering the time between spectral collection, adjust the spectrophotometer software to collect an absorbance spectrum every 50 mV. Do not start the absorbance measurement.

*Sample parameters:*

Collect one spectrum every 2.5 s (at a scan rate of 20 mV/s, it takes 2.5 s to reach +0.05 mV)

Collect 40 spectra (the time required to collect (0.0 V 🡨 🡪 +1.0 V) at 20 mV/s is 100 s)

- Ensure that both the spectrophotometer and potentiostat control platforms are visible on the computer screen.
- Begin the cyclic voltammogram and promptly move the cursor to the spectrophotometer controls. When the 5 seconds of quiet time have passed, begin absorbance measurements.

1. Ensure all spectra and the cv are saved, and then proceed to data processing.

Questions for data processing and analysis

*Plot the P3DDT cv collected in Part A step 2, and overlay the spectra collected in Part A, steps 4-10. On the voltammogram, mark the potentials at which the P3DDT was poised prior just to collection of each absorbance spectrum. If you haven’t done so already, use these spectra to complete Table 3.*

*
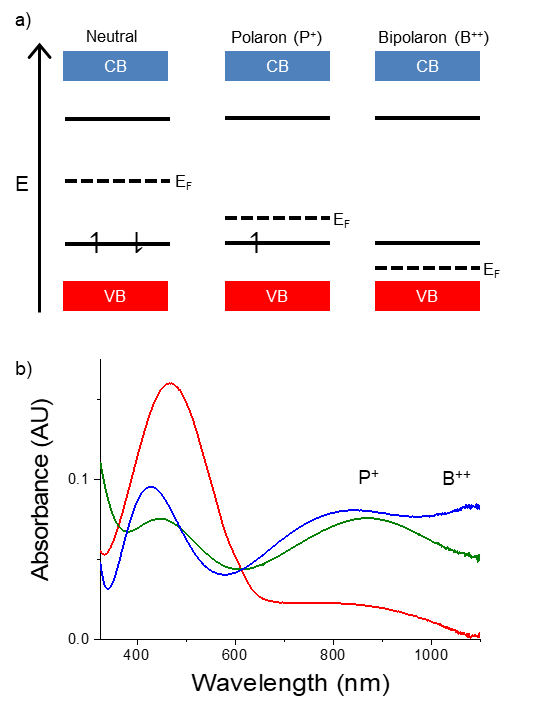
*

*Band diagrams for neutral, polaronic, and bipolaronic poly(alkylthiophene) are provided. On each band diagram, identify the electronic transition(s) observed in Part A. Using the spectra from Part A, determine the energy of each electronic transition in eV.*

**Module 3: Spectroelectrochemistry of poly(3-dodecylthiophene), continued**

*Using particle diagrams, depict the neutral, polaronic, and bipolaronic P3DDT subpopulations at the molecular level.*

*Overlay the spectra collected in Part B. Note that these are difference spectra, so the y-axis is in units of ‘change in absorbance,’ ΔA). If you haven’t done so already, use these spectra to complete Table 4. Note that a decrease in absorbance at a particular wavelength (-ΔA) means that the P3DDT subpopulation corresponding to the absorbance is decreasing in abundance, while an increase in absorbance (+ΔA) means that the P3DDT subpopulation corresponding to the absorbance is increasing in abundance.*

*How do these spectra compare to the spectra collected in Part A? What additional information can we learn from the spectra collected in Part B?*

*The neutral, polaronic, and bipolaronic P3DDT subpopulations exist simultaneously but vary in relative abundance. How might the relative abundance of a given subpopulation impact the properties of P3DDT relevant to sensors or other devices? For instance, would you expect the conductivity of the P3DDT to change as the subpopulation distribution changes? If so, describe.*

Note: For comparison, representative data obtained during Module 3 are given in Figure 3 and Figure 7 of the manuscript.

**Module 4: Analysis of CPMEs using solution redox-active species**

In this module, you will investigate how P3DDT modification of the WE impacts the electron transfer processes for three redox-active probe molecules. As controls, you will collect cyclic voltammograms for the probes (decamethyferrocene, ferrocene, and TPD) on ITO WEs, and you will collect cyclic voltammograms for the P3DDT in the potential windows relevant to the probes. This will allow you to distinguish the impacts of the P3DDT on the redox probe from other signals, enabling more clear interpretation of the cv’s for the probes collected on the P3DDT-modified WEs.

**Learning Outcomes**

*After completion of this module, you will be able to…*

- Use solution redox probes to examine the CPME conductivity as a function of PAT subpopulation
- Realize it is possible to alter the relative concentrations of neutral and charged PAT subpopulations electrochemically to meet design criteria in a desired application.

Use Tables 5 and 6 to record observations during data and after collection. Think through and briefly answer the questions embedded in the procedure during data collection. Use the question at the end of the module for more thorough data processing and analysis.

Table 5: Analysis of redox probes using an ITO WE

| Probe | *E_p_*_, ox_ (V) | *E_p_*_, red_ (V) | *ΔE (V)* | *i_p_*_, ox_ (A) | *i_p_*_, red_ (A) |
| --- | --- | --- | --- | --- | --- |
| Me_10_Fc |  |  |  |  |  |
| Fc |  |  |  |  |  |
| TPD |  |  |  |  |  |

Table 6: Analysis of redox probes using an P3DDT WE*

| Probe | *E_p_*_, ox_ (V) | *E_p_*_, red_ (V) | *ΔE (V)* | *i_p_*_, ox_ (A) | *i_p_*_, red_ (A) |
| --- | --- | --- | --- | --- | --- |
| Me_10_Fc |  |  |  |  |  |
| Fc |  |  |  |  |  |
| TPD |  |  |  |  |  |

*If both P3DDT and probe features are present in a given cv, record values for the peals corresponding to the probe molecule.

**Module 4: Analysis of CPMEs using solution redox-active species, continued**

**Procedure***

- Prepare 6 ITO CEs, 3 ITO WEs, and 3 functionalized ITO WEs
- Prepare 100 mL of the electrolyte solution: 0.1 M TBAPF_6_ (acetonitrile)
- Prepare 25 mL of the monomer solution using some of the prepared electrolyte solution to yield a solution that is 0.05 M 3DDT and 0.1 M TBAPF_6_ in acetonitrile
- Prepare 10 mL of the ferrocene (Fc) solution using some of the prepared electrolyte solution to yield a solution that is 1 mM Fc and 0.1 M TBAPF_6_ in acetonitrile
- Prepare 10 mL of the decamethylferrocene (Me_10_Fc) solution using some of the prepared electrolyte solution to yield a solution that is 1 mM Me_10_Fc and 0.1 M TBAPF_6_ in acetonitrile
- Prepare 10 mL of the *N*,*N*’-*bis*(3-methylphenyl)-*N*,*N*’-diphenylbenzidine (TPD) solution using some of the prepared electrolyte solution to yield a solution that is 1 mM TPD and 0.1 M TBAPF_6_ in acetonitrile
- Using the procedure outlined in Module 1, Part B, electrodeposit three P3DDT samples, all with a charge density of -4x10^-3^ C/cm^2^. Rinse each P3DDT sample with acetonitrile, dry each sample with a stream of gaseous nitrogen, and set these samples aside for later use.

*All potentials are with respect to an Ag|Ag^+^ nonaqueous RE (10 mM AgNO_3_, 0.1 M TBAPF_6_ in acetonitrile).

Part A: Decamethylerrocene

1. Assemble the electrochemical cell with a fresh ITO CE, a P3DDT-modified ITO WE, and the RE. Fill the cell with electrolyte solution.
2. Record a cyclic voltammogram using the following parameters: potential range -0.8 V to -0.1 V vs. Ag|Ag^+^, 1 cycle, scan rate = 50 mV/s.

*Is the P3DDT electrochemically active in this potential window? How might this impact the electron transfer processes of Me_10_Fc at the P3DDT-modified WE?*

1. Taking care not to disturb the WE, remove the electrolyte solution using a pipette, and refill the cell with Me_10_Fc solution.
2. Record a cyclic voltammogram using the following parameters: potential range -0.8 V to -0.1 V vs. Ag|Ag^+^, 1 cycle, scan rate = 50 mV/s.
3. Rinse the cell thoroughly, and reassemble the electrochemical cell with a fresh ITO CE, an ITO WE, and the RE. Fill the cell with dmFc solution.
4. Record a cyclic voltammogram using the following parameters: potential range -0.8 V to -0.1 V vs. Ag|Ag^+^, 1 cycle, scan rate = 50 mV/s.
5. Thoroughly rinse the electrochemical cell and all electrodes with acetonitrile before proceeding to the next part.

*What differences to you notice when comparing the Me_10_Fc cv collected on the P3DDT-modified WE to the cv of the same probe molecule on an ITO WE? Suggest chemical explanations for differences observed.*

**Module 4: Analysis of CPMEs using solution redox-active species, continued**

Part B: Ferrocene

1. Assemble the electrochemical cell with a fresh ITO CE, a P3DDT-modified ITO WE, and the RE. Fill the cell with electrolyte solution.
2. Record a cyclic voltammogram using the following parameters: potential range -0.2 V to +0.7 V vs. Ag|Ag^+^, 1 cycle, scan rate = 50 mV/s.

*Is the P3DDT electrochemically active in this potential window? How might this impact the electron transfer processes of Fc at the P3DDT-modified WE?*

1. Taking care not to disturb the WE, remove the electrolyte solution using a pipette, and refill the cell with Fc solution.
2. Record a cyclic voltammogram using the following parameters: potential range -0.2 V to +0.7 V vs. Ag|Ag^+^, 1 cycle, scan rate = 50 mV/s.
3. Rinse the cell thoroughly, and reassemble the electrochemical cell with a fresh ITO CE, an ITO WE, and the RE. Fill the cell with the Fc solution.
4. Record a cyclic voltammogram using the following parameters: potential range -0.2 V to +0.7 V vs. Ag|Ag^+^, 1 cycle, scan rate = 50 mV/s.
5. Thoroughly rinse the electrochemical cell and all electrodes with acetonitrile before proceeding to the next part.

*What differences to you notice when comparing the Fc cv collected on the P3DDT-modified WE to the cv of the same probe molecule on an ITO WE?*

*Were these cv’s different in the ways you expected, based on the differences between the dmFc cv’s as a function of WE? Explain.*

Part C: TPD

1. Assemble the electrochemical cell with a fresh ITO CE, a P3DDT-modified ITO WE, and the RE. Fill the cell with electrolyte solution.
2. Record a cyclic voltammogram using the following parameters: potential range +0.1 V to +0.9 V vs. Ag|Ag^+^, 1 cycle, scan rate = 50 mV/s.

*Is the P3DDT electrochemically active in this potential window? How might this impact the electron transfer processes of TPD at the P3DDT-modified WE?*

1. Taking care not to disturb the WE, remove the electrolyte solution using a pipette, and refill the cell with TPD solution.
2. Record a cyclic voltammogram using the following parameters: potential range +0.1 V to +0.9 V vs. Ag|Ag^+^, 1 cycle, scan rate = 50 mV/s.

**Module 4: Analysis of CPMEs using solution redox-active species, continued**

1. Rinse the cell thoroughly, and reassemble the electrochemical cell with a fresh ITO CE, an ITO WE, and the RE. Fill the cell with the Fc solution.
2. Record a cyclic voltammogram using the following parameters: potential range +0.1 V to +0.9 V vs. Ag|Ag^+^, 1 cycle, scan rate = 50 mV/s.
3. Thoroughly rinse the electrochemical cell and all electrodes with acetonitrile.

*Why do the peak currents corresponding to TPD oxidation and reduction increase when using the P3DDT WE relative to the ITO WE? Hint – consider the AFM images collected in Module 2.*

Additional Questions

*Generate the following graphs:*

- *One graph with all cv’s collected in the -0.8 V to -0.1 V potential window (dmFc) plotted on a single set of axes*
- *One graph with all cv’s collected in the -0.2 V to +0.7 V potential window (Fc) plotted on a single set of axes*
- *One graph with all cv’s collected in the +0.1 V to +0.9 V potential window (TPD) plotted on a single set of axes*

*If you haven’t done so already, use these graphs to complete Tables 5 and 6. If a particular peak is not present in a given cv, just leave the corresponding cell blank on the table.*

*Describe how the electroactivity of the P3DDT and the redox behavior of the probe molecule on the P3DDT WE correlate. To do so, answer these types of questions:*

- *Is the probe electron transfer process observable on P3DDT?*
- *Is the probe electron transfer process different kinetically (different in ΔE) on P3DDT relative to ITO?*
- *When the P3DDT is not electrochemically active in the potential window for a probe, does this impact the probe redox behavior on P3DDT?*

*Briefly summarize any correlations observed.*

*Think back to the neutral, polaronic, and bipolaronic P3DDT subpopulations examined in Module 3, and explain how the relative abundances of these subpopulations impact the ways P3DDT functions as an electrode-modifier in devices.*

Note: For comparison, representative data obtained during Module 4 are given in Figure 5 of the manuscript.

**Rubrics for assessment of learning outcomes**

| **Goal 1: Students will learn and understand laboratory and characterization techniques ^a, b^** | | | |
| --- | --- | --- | --- |
| **Goal Components** | **Module Activity** | **Proficiency Standard(s)** | **Demonstrated?**  **(Yes or No)** |
| 1.1 Electrochemically fabricate conducting polymer modified electrodes (CPMEs) | Make solutions, assemble cell, operate potentiostat | CPME fabricated |  |
| 1.2 Characterize CPMEs using cyclic voltammetry | Operate potentiostat to collect cyclic voltammogram | Cyclic voltammogram collected and interpreted – Faradaic processes observed and identified as such |  |
| 1.3 Characterize CPMEs using AFM (topography) | Operate AFM to evaluate topography of CPME | Topography image collected and interpreted – surface roughness and dimensions of features observed |  |
| 1.4 Characterize CPMEs using absorbance spectroscopy | Operate UV-Vis spectrophotometer to acquire spectra of CPME | Spectra collected – appropriate blanks used, appropriate wavelength range used, reasonable S/N |  |
| 1.5 Characterize CPMEs using redox-active probe molecules in solution | In-lab and post-lab questions (Module 4) | Differences between voltammograms on CPME only, redox-active species on an ITO WE, and redox active species on a CPME observed and identified |  |

^a^ Assessment of MS students and undergraduates preparing for independent research: Demonstration of proficiency was required for all goal components and was readily accomplished.

^b^ Suggested assessment of undergraduate students: Adapt rubric to reflect the modules completed in the local setting. Require demonstration of proficiency on Goal Components 1.1 and 1.2 to earn a passing grade.

**Rubrics for assessment of learning outcomes, continued**

| **Goal 2: Students will learn how to develop chemically rational explanations for/from measured signals ^a, b^** | | | |
| --- | --- | --- | --- |
| **Goal Components** | **Module Activity** | **Proficiency Standard(s)** | **Demonstrated?**  **(Yes or No)** |
| 2.1 Explain electrochemical signals observed from molecular-level perspectives, clearly articulating the processes that give rise to the measured signals in the context of electropolymerization, electrodeposition, and behaviors as a function of polymer subpopulations | In-lab and post-lab questions (Module 1) | Relate Faradaic current to polymer processes and subpopulations |  |
| 2.2 Relate electrochemical data (current density, charge density) to the amount of polymer electrodeposited | In-lab and post-lab questions (Module 1) | Increased current density (or charge density) during electropolymerization yields increased amounts of electrodeposited polymer. |  |
| 2.3 Connect electrochemical data and topographical images to construct molecular-level understanding of polymer nucleation and growth processes from evidence | In-lab and post-lab questions (Module 2) | Relate increases in dimensions of features on topographical images to growth from nucleation sites; construct particle representation that matches evidence obtained |  |

^a^ Assessment of MS students and undergraduates preparing for independent research: Demonstration of proficiency was required for all goal components.

^b^ Suggested assessment of undergraduate students: To earn an A, demonstrate proficiency on all goal components. To earn a B, demonstrate proficiency on Goal Components 2.1 and 2.2 only; to earn a C, demonstrate proficiency on Goal Component 2.1 only. To earn a D, attempt to demonstrate proficiency on 1 or more goal components using inappropriate data.

**Rubrics for assessment of learning outcomes, continued**

| **Goal 3: Students will learn to evaluate and leverage structure-property relationships in electroactive polymers ^a, b^** | | | |
| --- | --- | --- | --- |
| **Goal Components** | **Module Activity** | **Proficiency Standard(s)** | **Demonstrated?**  **(Yes or No)** |
| 3.1 Use spectroscopic and spectroelectrochemical data to identify and better-understand neutral and charged polymer subpopulations | In-lab and post-lab questions (Module 3) | Relate electrochemical signals to spectroscopic signals; relate signals to polymer subpopulations |  |
| 3.2 Use solution redox probes to examine the CPME conductivity as a function of PAT subpopulation | In-lab and post-lab questions (Module 4) | Explain presence (or absence) of Faradic activity from redox-active species when the WE is a CPME using understanding of the polymer subpopulations present in a given potential window. |  |
| 3.3 Realize they can alter the relative concentrations of neutral and charged PAT subpopulations electrochemically to meet design criteria in a desired application. | In-lab and post-lab questions (Module 4) | Discuss ideal subpopulation distribution for a specific application. |  |

^a^ Assessment of MS students and undergraduates preparing for independent research: Demonstration of proficiency on Goal Component 3.1 was required. Attempts to achieve proficiency on Goal Components 3.2 and 3.3 required, but demonstration of proficiency not required.

^b^ Suggested assessment of undergraduate students: To earn an A, demonstrate proficiency on Goal Component 3.1 and attempt to demonstrate proficiently on Goal Components 3.2 and 3.3 using the appropriate dataset. To earn a B, attempt to demonstrate proficiency on all goal components using the appropriate datasets. To earn a C, attempt to demonstrate proficiency on Goal Component 3.1 using the appropriate datasets. To earn a D, attempt to demonstrate proficiency on 1 or more goal components using inappropriate data.

**References**

1. Heinze, J.; Frontana-Uribe, B. A.; Ludwigs, S., Electrochemistry of Conducting Polymers—Persistent Models and New Concepts. *Chem. Rev.* **2010,** *110* (8), 4724-4771.

2. Heinze, J.; Rasche, A.; Pagels, M.; Geschke, B., On the origin of the so-called nucleation loop during electropolymerization of conducting polymers. *J. Phys. Chem. B* **2007,** *111* (5), 989-997.

3. Elgrishi, N.; Rountree, K. J.; McCarthy, B. D.; Rountree, E. S.; Eisenhart, T. T.; Dempsey, J. L., A Practical Beginner’s Guide to Cyclic Voltammetry. *J. Chem. Educ.* **2018,** *95* (2), 197-206.

4. Jenkins, J. L.; Lee, P. A.; Nebesny, K. W.; Ratcliff, E. L., Systematic electrochemical oxidative doping of P3HT to probe interfacial charge transfer across polymer-fullerene interfaces. *J. Mater. Chem. A* **2014,** *2* (45), 19221-19231.

5. Luscombe, C. K.; Maitra, U.; Walter, M.; Wiedmer, S. K., Theoretical background on semiconducting polymers and their applications to OSCs and OLEDs. *Chemistry Teacher International* **2021**.
